# Focal adhesions contain three specialized actin nanoscale layers

**DOI:** 10.1038/s41467-024-46868-7

**Published:** 2024-03-21

**Authors:** Reena Kumari, Katharina Ven, Megan Chastney, Shrikant B. Kokate, Johan Peränen, Jesse Aaron, Konstantin Kogan, Leonardo Almeida-Souza, Elena Kremneva, Renaud Poincloux, Teng-Leong Chew, Peter W. Gunning, Johanna Ivaska, Pekka Lappalainen

**Affiliations:** 1 HiLIFE Institute of Biotechnology, University of Helsinki, FI-00014 Helsinki, Finland; 2 Turku Bioscience Centre, University of Turku and Åbo Akademi University, FI-20520 Turku, Finland; 3 Advanced Imaging Center, HHMI Janelia Research Campus, Ashburn, VA 20147 USA; 4 Faculty of Biological and Environmental Sciences, University of Helsinki, Helsinki, Finland; 5 Institut de Pharmacologie et de Biologie Structurale, Université de Toulouse, CNRS, UPS, Toulouse, France; 6 School of Biomedical Sciences, UNSW Sydney, Wallace Wurth Building, Sydney, NSW 2052 Australia; 7 Department of Life Technologies, University of Turku, FI-20520 Turku, Finland; 8 InFLAMES Research Flagship Center, University of Turku, Turku, Finland; 9 Foundation for the Finnish Cancer Institute, Tukholmankatu 8, FI-00014 Helsinki, Finland

**Keywords:** Actin, Focal adhesion, Microtubules, Mesenchymal migration

## Abstract

Focal adhesions (FAs) connect inner workings of cell to the extracellular matrix to control cell adhesion, migration and mechanosensing. Previous studies demonstrated that FAs contain three vertical layers, which connect extracellular matrix to the cytoskeleton. By using super-resolution iPALM microscopy, we identify two additional nanoscale layers within FAs, specified by actin filaments bound to tropomyosin isoforms Tpm1.6 and Tpm3.2. The Tpm1.6-actin filaments, beneath the previously identified α-actinin cross-linked actin filaments, appear critical for adhesion maturation and controlled cell motility, whereas the adjacent Tpm3.2-actin filament layer beneath seems to facilitate adhesion disassembly. Mechanistically, Tpm3.2 stabilizes ACF-7/MACF1 and KANK-family proteins at adhesions, and hence targets microtubule plus-ends to FAs to catalyse their disassembly. Tpm3.2 depletion leads to disorganized microtubule network, abnormally stable FAs, and defects in tail retraction during migration. Thus, FAs are composed of distinct actin filament layers, and each may have specific roles in coupling adhesions to the cytoskeleton, or in controlling adhesion dynamics.

## Abstract (web summary)

Focal adhesions are dynamic structures that link the cell to the extracellular matrix. Here, the authors report that focal adhesions contain tropomyosin-decorated actin filaments, and show evidence that suggests specific functions in adhesion dynamics and cell migration.

## Introduction

Interplay between cells and extracellular matrix (ECM) is critical for a range of physiological and pathological processes. For example, in embryonic development and during immune responses in adult tissues, cells must sense both chemical and mechanical properties of their environment for decision-making in proliferation, migration, and differentiation. The most prominent structures that link animal cells to ECM are focal adhesions (FAs). These complex protein assemblies interact with ECM through transmembrane integrin adhesion receptors, which are connected to the actin cytoskeleton through a large number of associated proteins. Defects in the architecture or regulation of FAs are associated with several pathological states, such as cancer metastasis and immunological disorders^1–5^.

At the leading edge of migrating mesenchymal cells, small (diameter < 250 nm) *nascent adhesions* form within the actin-rich lamellipodia. A subset of *nascent adhesions* undergoes maturation to *focal complexes* at the lamellipodium-lamella interface, and can further mature to *focal adhesions* in response to increased contractile forces from the associated actomyosin bundles, called stress fibers, or through externally applied forces to the cell^2,6,7^. Elevated traction forces also inhibit elongation of the FA –associated stress fibers to further increase the contractile force exposed to the adhesion^8^. Hence, FA assembly and maturation are tightly regulated by the organization of the actin cytoskeleton. However, if the actin cytoskeleton also contributes to other aspects of adhesion dynamics remains poorly defined.

In addition to assembly, the disassembly of FAs must also be tightly regulated. This is especially important during directional cell migration, where adhesion disassembly occurs towards the cell rear to allow tail retraction for movement^9^. The precise molecular mechanisms of adhesion disassembly are incompletely understood, but were shown to involve endocytosis of adhesion-proximal integrins and local proteolysis of ECM components^2,10,11^. Moreover, targeting microtubules to FAs has for long been known to trigger adhesion disassembly^12–15^. KANK family proteins, which interact with talin through their Kank amino-terminal (KN) domain, were identified as critical factors for microtubule-dependent disassembly of adhesions^16,17^. Also spectraplakin family protein AFC7/MACF1 is an important regulator of FA dynamics by cross-linking microtubule plus-ends and actin filaments, as well as interacting with several other proteins^18,19^. At the molecular level, association of microtubule plus-ends with FAs promotes adhesion turnover, at least partly by trapping RhoA GEF-H1 to microtubules, and hence inhibiting the local activation of RhoA^20^. Furthermore, microtubules deliver autophagosomes to mature FAs to promote their disassembly^21–23^. Therefore, the spatio-temporal interplay between microtubules and FAs is a complex process, which is also likely to involve other, currently unidentified components. Indeed, proteomics studies provided evidence that adhesions harbour hundreds of different proteins^24–28^, but the functions of only fraction of these have been identified so far.

A major breakthrough in understanding FA architecture came with the use of super-resolution interferometric photo-activated localization microscopy (iPALM), revealing that FAs are composed of at least three separate layers relative to the plasma membrane: (1). ‘Integrin signalling layer’ at the bottom of adhesions composed of transmembrane integrins, FAK, and paxillin, (2). ‘Force-transduction layer’, composed of vinculin and extended talin molecules, which link integrins to actin filaments, and (3). Uppermost ‘Actin-regulatory layer’, containing α-actinin cross-linked actin filaments and actin polymerizing factor VASP^3–5,29^. However, actin filaments within adhesions display wider vertical distribution compared to that of α-actinin, hinting at the presence of other, currently unidentified actin filament structures in FAs^30^. Moreover, in addition to VASP, several other actin filament assembly factors were shown to associate with focal adhesions^23,31–33^. Thus, the presence of a large number of actin-regulatory and signalling proteins, together with the need to precisely control FA assembly, maturation and disassembly during various cellular processes, suggests that the molecular organization of FAs may be more complex than depicted in current models.

## Results

### Tpm1.6 and Tpm3.2 generate specific actin filament layers along the vertical axis of focal adhesions

A key feature of actin filaments is their ability to assemble into functionally distinct arrays in cells^34–38^. Tropomyosins (Tpms) are ubiquitous actin-binding proteins, which have evolved to specify the functional diversity of cellular linear actin filament arrays^39,40^. The coiled-coil tropomyosin dimers form head-to-tail polymers along the major groove of actin filaments, and are only known to be functional when bound to actin filaments^41,42^. The four *tropomyosin* genes in mammals, *TPM1*, *TPM2*, *TPM3* and *TPM4*, can generate >40 Tpm isoforms through alternative splicing, and the different isoforms provide specific functional features to the associated actin filaments. This is because Tpm head-to-tail polymers control interactions of other proteins, such as myosins and ADF/cofilins, to actin filaments in a Tpm isoform-specific manner^43–46^. Several Tpm isoforms localize to actin stress fibers and regulate their assembly. From these, at least two isoforms, Tpm1.6 and Tpm3.2, appear to also co-localize with FAs^47^. Importantly, Tpm1.6 and Tpm3.2 follow slightly different ‘paths’ along the major groove of actin filament, and cannot co-polymerize with each other on the same actin filament. Moreover, both isoforms compete with α-actinin, the key component of the previously identified ‘actin-regulatory layer’ of FAs, for actin filament binding^44,48,49^. Thus, if Tpm1.6 and Tpm3.2 are indeed FA components, they may specify previously unknown, functionally distinct actin filament structures.

**Fig. 1:**
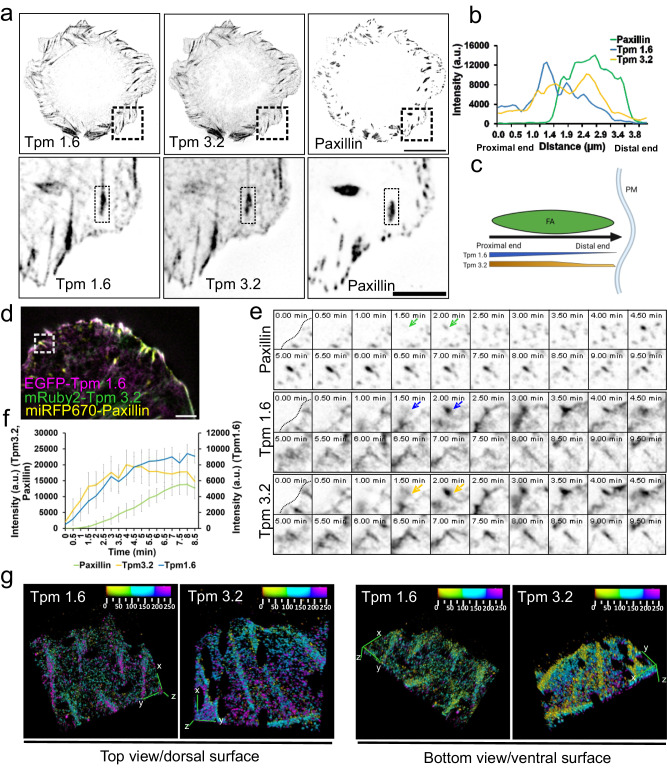
Tropomyosin-1.6 and tropomyosin-3.2 are early components of focal adhesions. **a** Representative TIRF images of wild-type U2OS cells expressing pmRuby2C1-Tpm1.6 and pEGFPC1-Tpm3.2, and stained for endogenous paxillin. The panels at the bottom are magnified images of the regions indicated with dashed boxes highlighting the localization of Tpm1.6 and Tpm3.2 in focal adhesions. Scale bars, 20 µm and 5 µm in upper and bottom rows, respectively. Experiments were repeated three times. **b** Line scan intensity profiles across the focal adhesion (from panel ‘**a**’). **c** Schematic representation of lateral localizations of Tpm1.6 and Tpm3.2 from the proximal to distal end of focal adhesions (see also Supplementary Fig. 1b). Created with BioRender.com. **d** TIRF image from a time-lapse movie of a wild-type U2OS expressing miRFP670-Paxillin, pEGFPC1-Tpm1.6 and pmRuby2C1-Tpm3.2. Scale bar, 50 µm. Experiment was repeated three times. **e** Individual channels of selected time-lapse frames from the magnified region (indicated by a white box in ‘**d**’). Black dotted lines indicate the cell edge. Green, purple and yellow arrows highlight the timing of paxillin, Tpm1.6 and Tpm3.2 accumulation to the focal adhesion, respectively. **f** Intensity profile analysis (*n* = 5 adhesions), demonstrating that paxillin intensity gradually increases in adhesions, whereas Tpm1.6 and Tpm3.2 intensities typically increase at focal adhesions more rapidly. However, the intensity dynamics of Tpm1.6 and Tpm3.2 are rather complex, and displayed substantial variation between different adhesions analyzed. The graph represents mean ± SE. **g** 3D-top view (image orientation from dorsal plane) and 3D-bottom view (image orientation from ventral plane) of rendered iPALM images of Tpm1.6 and Tpm3.2 from Supplementary Movie 2. The images were obtained by using ‘3D viewer’ plugin from FIJI/ImageJ. Note the enrichment of Tpm1.6 at the dorsal side (in top-view) and enrichment of Tpm3.2 at the ventral side (in bottom-view). The color-coding in the graph from red to purple shade represents the Z-depth from the coverslip (0 nm) towards the top of the focal adhesion (250 nm).

To investigate Tpm1.6 and Tpm3.2 localization in FAs, we employed total internal reflection fluorescence microscopy (TIRFM) imaging to detect structures localizing immediately adjacent to the cell-ECM interface. Using specific antibodies^50^ and fluorescent fusion proteins, we detected Tpm1.6 and Tpm3.2 localizing to FAs (Fig. 1a; Supplementary Fig. 1a), in agreement with a previous study using wide-field microscopy^47^. Tpm1.6 was distributed across the entire length of the adhesion and extended to the associated dorsal stress fiber, whereas Tpm3.2 localization was predominantly restricted to the adhesion (Fig. 1a–c; Supplementary Fig. 1b). Both tropomyosins partially co-localized with α-actinin-1 in adhesion (Supplementary Fig. 1c, d). Please note that the pronounced localization of α-actinin-1 to the distal ends of FAs is most likely due to enrichment of α-actinin-1 at the lamellipodial actin network^51^. Fluorescence-recovery-after-photobleaching (FRAP) analysis demonstrated that both tropomyosins are relatively stable components of FAs. However, consistent with earlier in vitro work on purified proteins^44^, Tpm3.2 displayed more rapid association-dissociation dynamics in FAs compared to Tpm1.6 (Supplementary Fig. 1e, f). Moreover, TIRF live-cell imaging on U2OS cells expressing EGFP-Tpm1.6, mRuby2-Tpm3.2, and miRFP670-Paxillin revealed that Tpm1.6 and Tpm3.2 were recruited to the newly forming adhesions approximately at the same time as paxillin. However, Tpm1.6 and Tpm3.2 displayed rather complex dynamics at adhesions with their intensities typically increasing rapidly over time, whereas paxillin intensity increased gradually at adhesions (Fig. 1d–f; Supplementary Movie 1). Thus, Tpm1.6 and Tpm3.2 are early components of FAs that display complex dynamics and slightly different lateral (xy) localization patterns with each other, and with α-actinin, in adhesions.

**Fig. 2:**
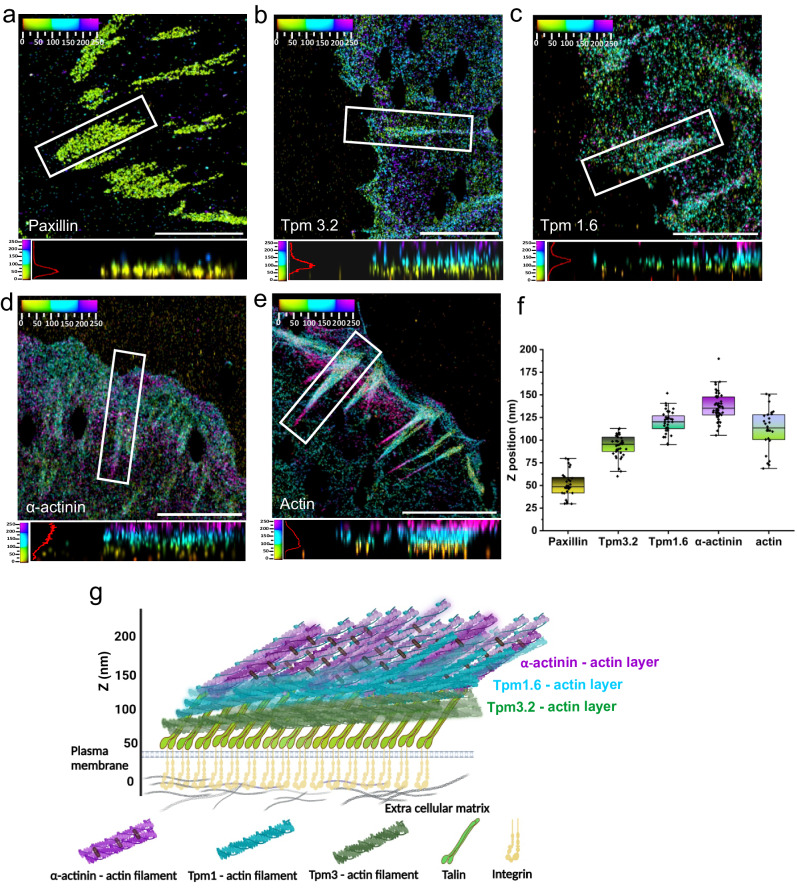
Tropomyosin-1.6 and tropomyosin-3.2 actin filament arrays form specific nanoscale layers in focal adhesions. **a**–**e** XY views and side views (indicated by white boxes in XY-view panels) from iPALM images of focal adhesions of U2OS cells. **a** Endogenous paxillin, **b** mEos3.2-Tpm3.2 (**c**) mEos3.2-Tpm1.6, (**d**) mEos3.2-α-actinin, and (**e**) endogenous actin detected with phalloidin. Scale bars, 5 µm. **f** Vertical stratification of focal adhesions located at the leading edge of the cell showing the Z-positions (Z_center_ +/− 1.5*IQR) of paxillin, Tpm3.2, Tpm1.6 and α-actinin, and F-actin. Each point in the graph corresponds to an individual focal adhesion measurement. Boxes display the mean, median, Whiskers, IQR: 25^th^–75^th^ percentiles, Whiskers range within 1.5*IQR. Please, see Supplementary Table 1 for iPALM statistics. **g** Schematic model of the molecular 3D architecture of a focal adhesion displaying the localizations of three ‘actin filament layers’ in the nanoscale strata. The positioning of each protein is based on the data presented in (**a**–**f**). The model does not depict the protein stoichiometry. Created with BioRender.com.

To examine the nanoscale localisation of Tpm1.6- and Tpm3.2-actin filaments in FAs, we applied iPALM, which allows 3D localization of tagged proteins with <20 nm accuracy^3–5,29,30^. First, we generated Eos-tagged Tpm1.6 and Tpm3.2 and validated their localization to FAs of U2OS cells together with α-actinin by TIRF imaging (Supplementary Fig. 1g). Interestingly, subsequent iPALM experiments provided evidence that Tpm1.6 and Tpm3.2 display distinct vertical localizations in respect to each other (Fig. 1g; Supplementary Fig. 2a, b; Supplementary Movie 2). To dissect the precise localization of Tpm1.6 and Tpm3.2 in the nanoscale strata of FAs, we used endogenous paxillin (visualized by antibody) as a reference marker for focal adhesions and the ‘integrin signalling layer’, and established photoactivatable fluorescent (PA-FP) protein of α-actinin-1^3,30^ as a reference protein for the ‘actin-regulatory layer’^3–5,30^. The vertical (*z*) localization of paxillin (Z_centre_ = 51 nm ± 14 nm) was consistent with its previously reported z-position in U2OS and other cell-types (Fig. 2a, f). The observed z-position of α-actinin was consistent with the previously reported z-position in cornerstone adhesions of pluripotent stem cells^30^, while slightly higher compared to the previous studies on U2OS and endothelial cells (see ‘Methods’ and Supplementary Table 1). The small variations may result from differences in the analysed adhesion types, or from the differences in coverslip coating for imaging.

To focus on similar adhesion types, we first analysed only those adhesions, which are located at the leading edge and oriented perpendicular to the cell edge (Supplementary Fig. 3a–e). Interestingly, our analysis revealed that the z-position of Tpm1.6 (Z_centre_ = 116 nm ± 13 nm) was slightly below the extended vertical distribution of α-actinin cross-linked actin filaments (Z_centre_ = 139 nm ± 22 nm), and displayed only a partial overlap with α-actinin (Fig. 2c, d, f). Moreover, the overall distribution range of Tpm1.6 (spanning ~25 nm) did not correspond to the wider z-distribution of α-actinin (spanning ~ 45 nm). Tpm3.2 localized to markedly lower vertical position (Z_centre_ = 92 nm ± 14 nm) compared to α-actinin and Tpm1.6, being thus positioned above paxillin at the ‘force transduction layer’ (Fig. 2b, c, f). Since the newly identified Tpm1.6- and Tpm3.2-actin arrays show distinct vertical nanoscale localizations in comparison to previously characterized α-actinin cross-linked actin filaments, we propose to call these newly identified actin layers as the ‘Tpm1.6-actin filament layer’ and ‘Tpm3.2-actin filament layer’ (Fig. 2f). Congruent with this notion, the vertical localization of actin filaments in U2OS cell adhesions displays a broad distribution, extending beyond the α-actinin positive layer and spanning all three layers (Tpm3.2, Tpm1.6, and α-actinin) identified in our iPALM experiments (Fig. 2e, f). In addition to FAs at the leading edge of cell, we analysed the vertical localizations of Tpm1.6 and Tpm3.2 in large, mature focal adhesions, which are associated with the ventral stress fibers and oriented more parallel along the cell edge. Similarly, Tpm1.6 and Tpm3.2 localized to two distinct vertical layers, as described above for the leading edge adhesions (Supplementary Fig. 2c–e). Finally, similar vertical localizations of Tpm1.6 and Tpm3.2 in FAs were also observed when these proteins were imaged by iPALM in the corresponding knockout cells lacking endogenous Tpm1.6 and Tpm3.2, respectively (Supplementary Fig. 2f). These latter experiments provide evidence that over-expression of Tpm1.6 or Tpm3.2 do not have drastic effects on the three-dimensional architecture of focal adhesions.

Together, these live-cell and super-resolution iPALM imaging experiments reveal that FAs are composed of at least three distinct actin-filament populations, which are specified by actin-binding proteins α-actinin, Tpm1.6 and Tpm3.2. Importantly, these actin filament populations display adjacent, but distinct localizations along the vertical axis of adhesions: the previously described α-actinin-actin at the top, followed by partially overlapping Tpm1.6-actin filaments and Tpm3.2-actin filaments slightly below (Fig. 2g).

### Tpm1.6 and Tpm3.2 have different roles in cell morphogenesis and migration

**Fig. 3:**
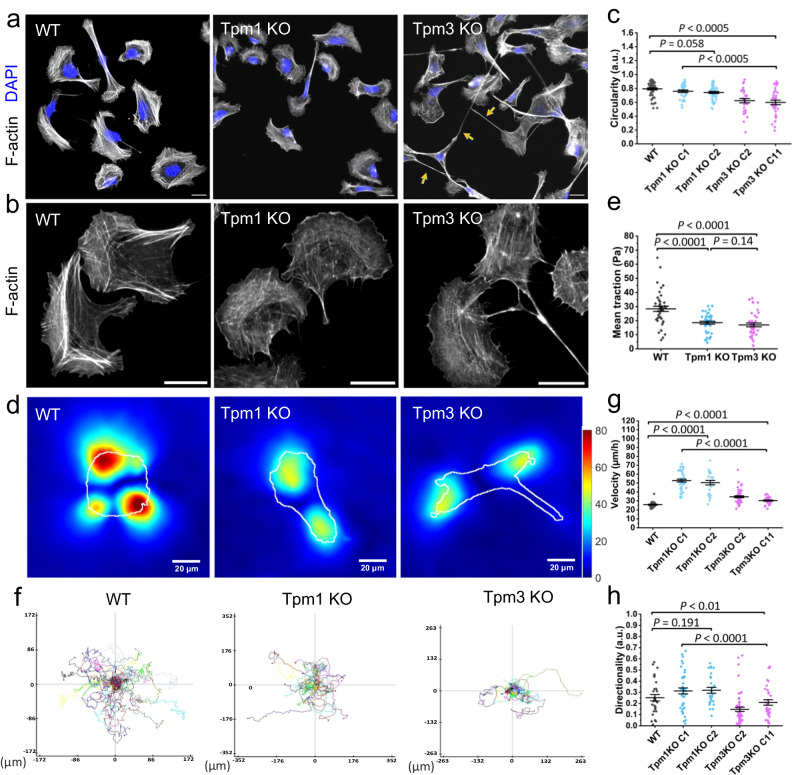
Effects of tropomyosin-1 and tropomyosin-3 depletions on cell morphology, force production, migration, and the actin cytoskeleton. **a** Representative wide-field images of wild-type, Tpm1 knockout, and Tpm3 knockout U2OS cells stained for F-actin (phalloidin) illustrating the morphological differences between the Tpm1 knockout and Tpm3 knockout cells. The arrows highlight the abnormal tails of the Tpm3 knockout cells. Scale bars, 20 µm. **b** Representative wide-field images of wild-type, Tpm1 knockout, and Tpm3 knockout cells stained for F-actin (phalloidin), demonstrating defects in the stress fiber networks of the Tpm1 knockout and Tpm3 knockout cells. Scale bars, 20 µm. **c** Cell circularity analysis of wild-type (*n* = 42), Tpm1 knockout; clone 1 (C1) (*n* = 44) clone 2 (C2) (*n* = 45), and Tpm3 knockout; clone 2 (C2) (*n* = 37) clone 11 (C11) (*n* = 38) cells after 90 min of plating. The data represents mean ± SE. The exact *p*-values (one-way ANOVA followed by Turkey multiple comparison test): *p* = 0.058; 0.00000003; 0.0000010. **d** Representative traction force maps of wild-type, Tpm1 knockout and Tpm3 knockout cells plated on 9.6 kPa hydrogels coated with fibronectin. Scale bar 20 µm. **e** Mean traction (per cell) of wild-type (*n* = 43), Tpm1KO (*n* = 41) and Tpm3KO (*n* = 42) cells on 9.6 kPa hydrogels coated with fibronectin. The data represents mean ± SE. The exact *p*-values (two-tailed Student’s *t* test): *p* = 0.000015; 0.0000035; 0.14. **f** Random migration trajectory maps of wild-type, Tpm1 knockout, Tpm3 knockout cells plated on fibronectin-coated substrate. Please, note the differences in the scales of x- and y-axes between wild-type, Tpm1 and Tpm3 panels. **g**, **h** Analysis of random migration rates and cell directionality of wild type (*n* = 30), Tpm1 knockout; clone 1 (C1) (*n* = 42) clone 2 (C2) (*n* = 27), and Tpm3 knockout; clone 2 (c2) (*n* = 55) clone 11 (C11) (*n* = 34) cells. The data represents mean ± SE. The exact *p*-values (one-way ANOVA followed by Turkey multiple comparison test) are provided in the Source data.

Considering the distinct localizations of Tpm1.6 and Tpm3.2-actin filaments in the nanoscale strata of FAs, we next determined the functions of these two actin-filament layers by generating knockout U2OS cell lines by CRISPR/Cas9. The obtained knockout clones were confirmed by Western blot, Sanger sequencing and Next Generation sequencing (Supplementary Fig. 4a–d). Please note that by targeting exon 1a of the *TPM1* gene, and exon 1b of the *TPM3* gene, we only depleted Tpm1.6 and the closely related Tpm1.7, and Tpm3.2 and the closely related Tpm3.1, respectively, from the Tpm isoforms expressed in U2OS cells (Supplementary Fig. 4a^52^); Depletions of Tpm1.6/7 (hereafter Tpm1 knockout cells) or Tpm3.1/2 (hereafter Tpm3 knockout cells) did not result in elevation or downregulation of the other isoform (Supplementary Fig. 4b). Both Tpm1 and Tpm3 knockout cells displayed thinner and less organized actin stress fibers compared to the wild-type cells (Fig. 3a, b; Supplementary Fig. 5c, d), in line with our previous siRNA studies^47^. This stress fiber phenotype was confirmed by mixing the Tpm1 KO and Tpm3 KO cells with wild-type U2OS cells stably expressing EGFP-CAAX, and comparing the organization of actin networks in control and knockout cells from the same images (Supplementary Fig. 4e). Consistent with the defects in the stress fiber networks, the Tpm1 and Tpm3 knockout cells generated diminished tractions forces (Fig. 3d, e) and displayed slightly reduced levels of active RhoA (Supplementary Fig. 4h). On the other hand, myosin II (both NMIIA and NMIIB) localization along stress fibers appeared largely unaffected in the Tpm1 and Tpm3 knockout cells (Supplementary Fig. 4i, j).

Although Tpm1 and Tpm3 depletions resulted in similar effects on stress fiber networks, the knockouts affected cell morphology and migration in different ways (Fig. 3a, b; Supplementary Fig. 4f). During the initial phase of cell spreading (90 min post plating) on a fibronectin-coated glass surface, wild-type and Tpm1 knockout cells displayed a relatively round and non-polarized morphology, whereas Tpm3 knockout cells were more elongated and polarized. These morphological differences were consistent in other Tpm1 and Tpm3 knockout clones (Fig. 3c) and were also retained after the Tpm1 and Tpm3 knockout cells were fully spread and polarized (8 h post plating) (Supplementary Fig. 4g). Interestingly, unlike wild-type and Tpm1 knockout cells, the majority of Tpm3 knockout cells exhibited abnormally long tails (Fig. 3a), suggesting that Tpm3 is critical for proper tail-retraction during cell migration. To explore this further, we examined random migration of cells plated on fibronectin. By tracking the movement of nuclei, we revealed that the Tpm1 knockout cells migrated with a higher velocity compared to Tpm3 knockout and wild-type U2OS cells (Fig. 3f, g; Supplementary Movie 3). On the other hand, Tpm3 knockout cells, but not Tpm1 knockout cells, displayed defects in directionality during migration (Fig. 3f–h; Supplementary Movie 3), and exhibited defective tail retraction (Fig. 3a; Supplementary Movie 3). Thus, Tpm1 and Tpm3 depletions result in opposite effects on cell morphology, polarity, and migration, suggesting that the Tpm1-actin and Tpm3-actin filament populations may regulate these processes via distinct molecular mechanisms.

### Opposite functions of Tpm1.6 and Tpm3.2 in spatio-temporal dynamics of focal adhesions

**Fig. 4:**
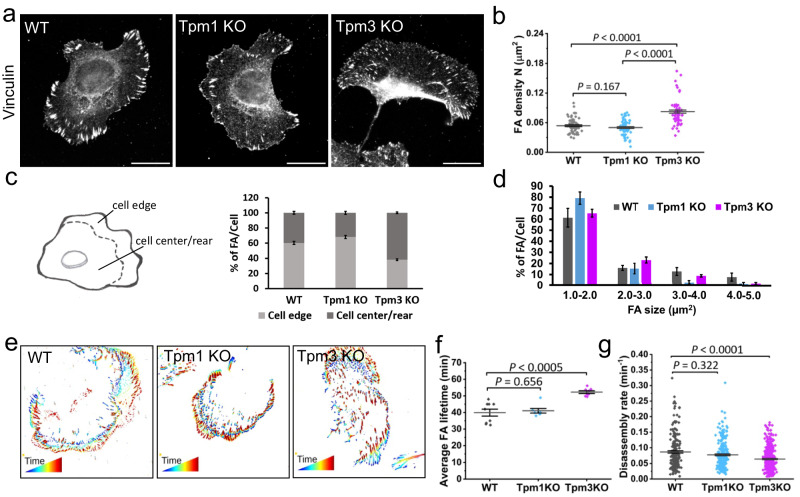
Depletions of tropomyosin-1 and tropomyosin-3 result in opposite effects on focal adhesion distribution and dynamics. **a** Representative wide-field images of wild-type, Tpm1 knockout, and Tpm3 knockout U2OS cells plated on fibronectin-coated coverslips and stained for focal adhesions (vinculin antibody). Scale bars, 20 µm. **b** Focal adhesion density analysis from wild-type (*n* = 70), Tpm1 knockout (*n* = 62), and Tpm3 knockout (*n* = 63) cells. The data represents mean ± SE. The exact *p*-values (two-tailed Student’s *t* test) are provided in the Source data. **c** Distributions of focal adhesions at the cell edge (within 5 μm from the leading edge) vs. cell centre/rear. Quantification of the percentage of focal adhesions located in these regions in wild-type (*n* = 30), Tpm1 knockout (*n* = 30) and Tpm3 knockout (*n* = 25) cells are shown in the graph. The data represents mean ± SE. **d** Quantification of the size distributions of focal adhesions in wild-type (*n* = 30), Tpm1 knockout (*n* = 30) and Tpm3 knockout (*n* = 25) cells. The data represents mean ± SE. **e** Representative examples of temporal color-coded TIRF microscopy time-lapse movies of miRF670-Paxillin expressing cells depicting the dynamic mode of focal adhesions (generated from FAAS). **f** Quantitative analysis of average focal adhesion lifetimes from TIRF microscopy time-lapse movies of miRFP670-Paxillin expressing wild-type (*n* = 9 cells), Tpm1 knockout (*n* = 7 cells), and Tpm3 knockout (*n* = 8 cells) cells. The data represents mean ± SE. The exact *p*-values (two-tailed Student’s *t* test): *p* = 0.656; 0.00023. **g** Analysis of focal adhesion disassembly rates calculated from TIRF microscopy time-lapse movies of miRFP670-Paxillin expressing cells by using FAAS. Wild-type (*n* = 9 cells), Tpm1 knockout (*n* = 7 cells), and Tpm3 knockout (*n* = 8 cells). The data represents mean ± SE. The exact p values (Mann-Whitney test): *p* = 0.322; 0.0000061.

The localisation of Tpm1.6-actin and Tpm3.2-actin to different actin filament ‘layers’ in FAs (Fig. 2g), and their distinct effects on cell morphology and migration (Fig. 3) suggest that these proteins may regulate the organization or dynamics of FAs by different mechanisms. The density of vinculin-positive FAs was significantly increased in the Tpm3 knockout cells compared to the wild-type cells, whereas the adhesion density was unaffected in the Tpm1 knockout cells (Fig. 4a, b; Supplementary Fig. 5e, f). Moreover, the Tpm1 knockout cells displayed an almost complete absence of large (area >3 μm^2^) adhesions, whereas the adhesion size-distribution was less affected by depletion of Tpm3 (Fig. 4d; Supplementary Fig. 5e, f). Perhaps most interestingly, Tpm1 and Tpm3 knockouts had opposite effects on the spatial distribution of adhesions in cells. In the Tpm1 knockout cells, the adhesions were smaller and predominantly localized at the leading edge (~70 % of adhesions were within 5 μm distance from the leading edge), whereas the majority (>60 %) of adhesions in Tpm3 knockout cells were located at the cell centre or cell rear (Fig. 4c).

Previous studies indicated that accumulation of nascent peripheral adhesions correlates with increased integrin-β1 activity^53^. This prompted us to probe for integrin-β1 activity in these cells. Freely migrating, unconfined Tpm1 knockout cells had significantly more active integrin-β1 positive adhesions, concordant with the higher migration velocity of these cells (Supplementary Fig. 6a, b). In contrast, the total active integrin-β1 intensity per cell was significantly lower in the Tpm3 knockout cells compared to the control cells (Supplementary Fig. 6c). To further distinguish whether the changes in integrin activity are a cause or a consequence of the different migratory properties of the Tpm1 and Tpm3 knockout cells, we confined cells to cross-bow micropatterns, which normalize cell shape and prevent cell motility. Integrin-β1 activity was higher in the micropattern-bound Tpm1 knockout cells, whereas there was no significant difference in integrin activity between the control and the Tpm3 knockout cells (Supplementary Fig. 6d, e). Thus, depletion of Tpm1 elevates integrin activity independently of cell migration, indicating that Tpm1 limits integrin activation in nascent adhesions, supporting their maturation at the leading edge. In contrast, Tpm3 depletion attenuates integrin activity only in unconfined cells implying that Tpm3 supports integrin activity in adhesions in a manner that is linked to turnover of already existing focal adhesions. To further explore the differences in integrin activity, we investigated the expression levels of established integrin activators (talin and kindlin-2 and ref. ^54^) and integrin-β1 inhibitor SHARPIN^55^ (Supplementary Fig. 6f, g). Talin expression was somewhat variable but not significantly different between the cells, whereas SHARPIN was modestly down-regulated in the Tpm3 knockout cells. Kindlin-2 protein was strongly downregulated in the Tpm3 knockout cells and to a lesser extent also in the Tpm1 knockout cells. The down-regulation of kindlin-2 could be functionally linked to lower integrin-β1 activity in the Tpm3 knockout cells. However, given the impact of confinement on the integrin activity, it is likely that integrin activity levels are determined by complex crosstalk of receptor activation states regulated by activating and inactivating proteins and adhesion dynamics governed by Tpm-actin filaments.

To determine if the differences in spatial distributions of FAs in Tpm1 and Tpm3 knockout cells are linked to altered adhesion dynamics, we performed TIRF live-cell imaging of wild-type, Tpm1 knockout, and Tpm3 knockout cells transiently expressing miRFP tagged paxillin (Fig. 4e; Supplementary Movies 4–6). Analysis of the obtained movies by Focal Adhesion Analysis Server (FAAS)^56,57^, demonstrated that the average lifetime of adhesions per cell was unaffected by the depletion of Tpm1, whereas average lifetimes of adhesions were increased by ~30% in the absence of Tpm3 (Fig. 4e, f). Analysis of the dynamics of individual paxillin-positive adhesions by FAAS also provided evidence that the FA disassembly rates were significantly diminished in the Tpm3 knockout cells, consistent with their increased adhesion lifetimes (Fig. 4g). Together, these data suggest that Tpm1.6-actin filament arrays may be important for FA maturation. On the other hand, Tpm3.2-actin filament arrays may be crucial for proper FA disassembly at the cell center and cell rear. Consequently, depletions of Tpm1 and Tpm3 lead to opposite effects in the subcellular distribution and dynamics of FAs.

### Tpm3.2 controls focal adhesion disassembly through KANK- and ACF7/MACF1-dependent microtubule-targeting

**Fig. 5:**
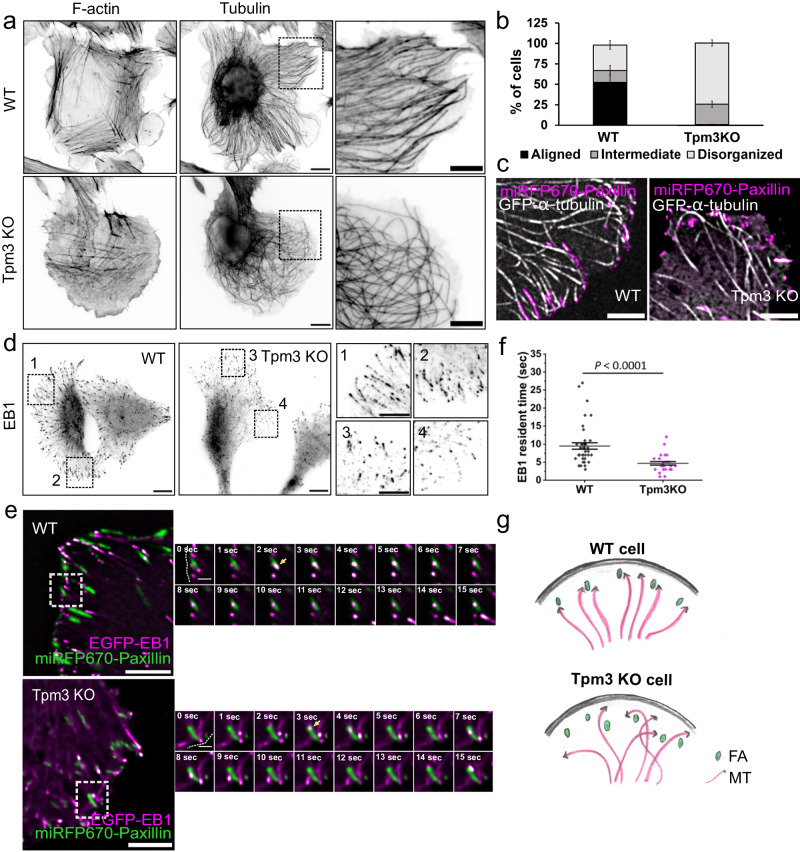
Tpm3 is critical for proper microtubule organization and focal adhesion targeting. **a** Wide-field images of wild-type and Tpm3 knockout U2OS cells stained for F-actin (phalloidin) and microtubules (α-tubulin antibody). The panels on the right are magnified images of the regions at the cell periphery indicated with black boxes in the whole cell images. Scale bars, 10 µm and 5 µm, respectively. Experiments were repeated three times. **b** Blinded analysis of the percentage of cells displaying aligned, intermediate and tangled microtubule networks in wild-type (*n* = 219) and Tpm3 knockout (*n* = 344) cells. The data are from three independent experiments and represent mean ± SE. **c** TIRF microscopy images of wild-type U2OS and Tpm3KO cells expressing miRFP670-Paxillin and GFP-α-tubulin demonstrating diminished targeting of microtubules to focal adhesions. Scale bars, 5 µm. **d** Wide-field images of wild-type and Tpm3 knockout U2OS cells stained for microtubules plus-end tracking protein EB1. The panels on the right (1-4) are magnified images of the regions indicated with black boxes in the whole cell images. Scale bars, 10 µm 5 µm, respectively. Experiments were repeated three times. **e** TIRF time-lapse images of wild-type and Tpm3 knockout cells expressing EGFP-EB1 and miRFP670-Paxillin. Selected time-lapse frames from the magnified areas (indicated by white boxes) are shown on the right as merged frames. Yellow arrows highlight the point of initial contact of EB1 with focal adhesions. Scale bars, 5 µm and 1 µm, respectively. **f** Analysis of the EB1 resident times in focal adhesions analysed from TIRF time-lapse movies of cells expressing EGFP-EB1 and miRFP670-Paxillin. Wild-type (*n* = 40 EB1 foci from 5 movies) and Tpm3 knockout (*n* = 26 EB1 foci from 5 movies) cells. The data represents mean ± SE. The exact *p*-value (two-tailed Student’s *t*-test): *p* = 0.000015. **g** Schematic representation of the organization of microtubule plus-ends in wild-type and in Tpm3 knockout cells based on the data presented here.

FA disassembly must be accurately regulated to maintain proper spatio-temporal distribution of adhesions for cell migration and morphogenesis. Microtubule targeting to focal adhesions is crucial for adhesion turnover, but the underlying molecular details are incompletely understood. Given the FA disassembly defects in the Tpm3 knockout cells, we examined the effects of Tpm3 depletion on the microtubule organization in interphase cells. Interestingly, compared to the wild-type and Tpm1 knockout U2OS cells, the Tpm3 knockout cells displayed microtubules with ‘criss-cross/tangled’ (disorganized) pattern. Moreover, the individual microtubules of Tpm3 knockout cells were less straight/aligned, and often bent at the cell periphery (Fig. 5a; Supplementary Fig. 7a). Blinded analysis of cells stained for α-tubulin revealed that whereas ~50% of wild-type cells had properly aligned microtubules organized in parallel arrays, such ‘normal’ organization of microtubules was not detected in the Tpm3 knockout cells. In contrast, >70% of Tpm3 knockout cells exhibited severely disorganized microtubule arrays characterized by bent microtubules, which formed a ‘criss-cross’ network (Fig. 5b). In addition to defects in the general organization of the microtubule networks, both the subcellular distribution and morphology of microtubule plus-end tracking protein EB1 foci were affected by Tpm3 depletion (Fig. 5d; Supplementary Fig. 7b). It is also important to note that defects in microtubule organization were detected only in the Tpm3 knockout cells, although both Tpm1 and Tpm3 depleted cells displayed comparable defects in actin stress fiber organization. Thus, the abnormal, ‘criss-cross/tangled’ microtubule pattern in the Tpm3 knockout cells is unlikely to arise from defects in stress fiber organization.

To examine the interplay between microtubules and FAs, we performed TIRF live-cell imaging on U2OS cells co-expressing GFP-α-tubulin and miRFP-paxillin, as well as on cells co-expressing EGFP-EB1 and miRFP-paxillin. Wild-type cells exhibited relatively stable association of microtubule plus-ends with FAs, as well as ‘poking behaviour’, where microtubules transiently targeted adhesions. In the Tpm3 knockout cells, however, microtubules were poorly targeted to FAs, and often bypassed adhesions and subsequently continued to elongate parallel to the cell edge before disassembling (Fig. 5c; Supplementary Movie 7). Furthermore, TIRF imaging revealed significantly shorter resident times of EB1 foci with FAs in Tpm3 knockout cells as compared to wild-type cells (Fig. 5e–g; Supplementary Movie 8).

**Fig. 6:**
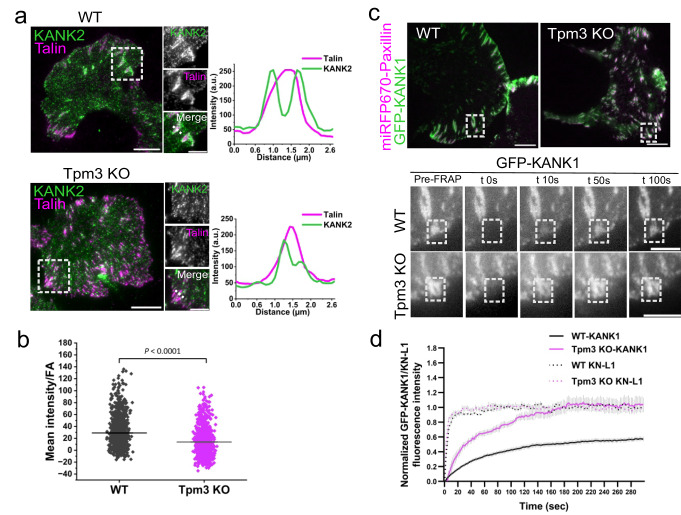
Tropomyosin-3 regulates microtubule – focal adhesion interactions by stabilizing KANK at focal adhesions. **a** TIRF images of wild-type and Tpm3 knockout cells stained for endogenous KANK2 and talin. The panels to the right are magnified images of the regions indicated with white boxes in the whole cell images. Scale bars, 10 µm and 5 µm, respectively. The intensity line scans on the right are from the selected adhesion regions demonstrating compromised localization of KANK2 at the rim of the focal adhesions in Tpm3 knockout cells. **b** Quantification of the mean intensity of endogenous KANK2 in talin positive FAs in cells plated on fibronectin-coated surface (*n* = 1036 adhesions for wild-type, and *n* = 855 adhesions for Tpm3 knockout cells). The data represent mean ± SE. The exact p value (two-tailed Student’s *t*-test) is provided in the Source data. Please note that the KANK2 intensity in FAs was compared to a randomly-selected adhesion-free area of the cytoplasm from the same cell, and thus the KANK2 intensity values of a subset of adhesions, especially in the Tpm3 knockout cells, were negative. **c** Representative examples of GFP-KANK1 time-lapse images (from the FRAP experiment) from wild-type and in Tpm3 knockout cells. Scale bars, 10 µm. The panels below are magnified images of the regions indicated with white boxes, and represent selected time-frames of the FRAP experiments. The time-point ‘Pre’ is a frame before bleaching and ‘0 s’ is the first frame after bleaching. The white boxes indicate the bleached regions. Scale bars, 5 µm. **d** Quantification of the fluorescence recovery of GFP-KANK1 and GFP-KN-L1 in FAs of wild-type and Tpm3 knockout cells. Graph shows mean curves ± S.E.M. over time. The measurements are from (*n* = 29 adhesions for KANK1 from 7 movies, *n* = 21 adhesions for KN-L1 from 6 movies) wild-type and (*n* = 38 adhesions for KANK1 from 6 movies, *n* = 21 adhesions for KN-L1 from 9 movies) Tpm3 knockout cells.

**Fig. 7:**
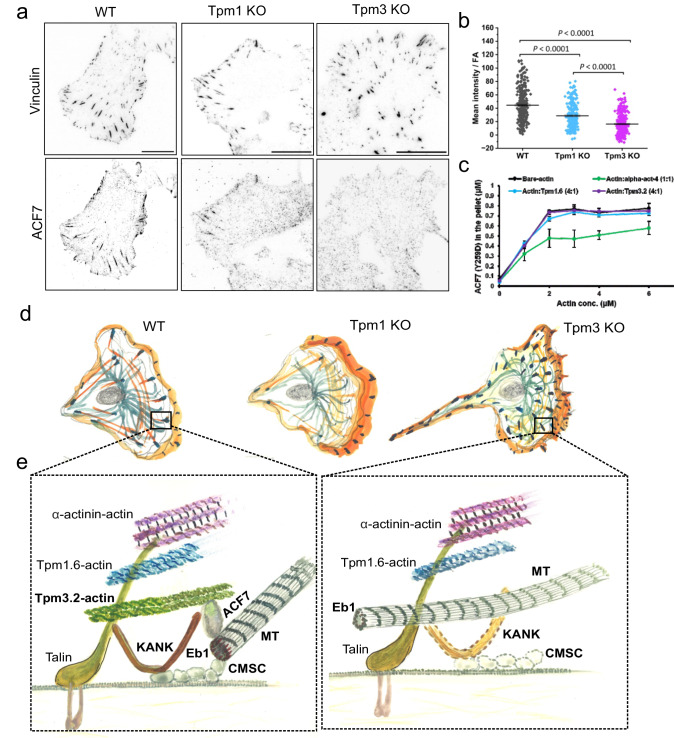
Tropomyosin-3 regulates microtubule – focal adhesion interactions by stabilizing ACF7 at focal adhesions. **a** TIRF images of wild-type, Tpm1 knockout, and Tpm3 knockout cells stained for endogenous vinculin and ACF7. Scale bars, 20 μm. **b** Quantification of ACF7 mean intensity in vinculin-positive adhesions from cells plated on fibronectin coated surface (*n* = 285 adhesions from 12 wild-type cells, *n* = 225 adhesions from 18 Tpm1 knockout cells, and n = 298 adhesions from 22 Tpm3 knockout cells). The data represent mean ± SE. The exact *p*-values (two-tailed Student’s *t* test) are provided in the Source data. **c** Co-sedimentation assay to measure binding of ACF7(73-306) fragment to bare β/γ-actin filaments, and to β/γ-actin filaments saturated with Tpm1.6, Tpm3.2, or α-actinin-4. The concentration of ACF7(73-306) was 1 µM. The amounts (µM) ACF7(73-306) in the pellet fractions (y-axis) in respect to concentration (µM) of actin (x-axis) are shown. Values = mean; error bars ± S.E.M (*n* = 3). **d** Schematic representation of polarized wild-type, Tpm1 knockout and Tpm3 knockout cells, displaying the spatial organization of focal adhesions (black), actin (orange) and microtubule networks (green). Tpm1 depletion leads to defects in focal adhesion maturation, whereas Tpm3 depletion results in defective targeting of microtubules to focal adhesions, and accompanied problems in adhesion disassembly and tail retraction during cell migration. **e** Schematic representation of microtubule-dependent FA disassembly in wild-type cells, where we propose that Tpm3.2-actin filaments (green) close to the bottom of adhesion stabilize KANK (orange) and ACF7, along with cortical microtubule stabilizing complex (CMSC), at adhesions. Thus, Tpm3.2-actin filaments may facilitate targeting of microtubule (MT, yellow) plus-ends to the adhesion. In the absence of Tpm3.2, ACF7 does not accumulate to FAs and stable association of KANK and cortical microtubule stabilizing complex (CMSC) components is lost at adhesions. This results in defective targeting of microtubule plus-end to the adhesion.

To elucidate the mechanism by which Tpm3.2–actin filaments mediate microtubule targeting to FAs, we focused on a multi-protein complex referred to as the ‘cortical-microtubule-stabilizing complex’ (CMSC), consisting of KANKs, ELKS, liprins, LL5β and CLASPs^58^, as well as of the spectraplakin family protein ACF7/MACF1^19^. Among the CMSC proteins, KANK directly binds to the core FA component talin. Two isoforms of this protein family, KANK1 and KANK2, localize to the rim of FAs and are critical factors in microtubule-dependent adhesion disassembly^16,20,59^. TIRF imaging revealed that endogenous KANK1 and KANK2 localized either to the adhesion periphery or in the close vicinity of FAs in wild-type cells, whereas in Tpm3 knockout cells these proteins appeared to display more diffuse distributions (Fig. 6a, b; Supplementary Fig. 8a, b; Supplementary Fig. 9a, b). iPALM imaging revealed that the C-terminus of KANK1 and Tpm3.2 displayed similar vertical positions in FAs of U2OS cells (Supplementary Fig. 9d, e). Next, we investigated the effects of Tpm3 depletion on the turnover of GFP-KANK1 at miRFP-paxillin labelled FAs using FRAP. GFP-KANK1 localized to miRFP-paxillin positive adhesions in the wild-type cells, and somewhat less extensively in the Tpm3 knockout cells (Fig. 6c). FRAP analysis revealed that GFP-KANK1 was quite stably associated with FAs in wild-type cells (with a stable fraction of ~50 %), whereas GFP-KANK1 displayed much more rapid dynamics in adhesions of Tpm3 knockout cells (with apparently no stable fraction) (Fig. 6c, d; Supplementary Fig. 9b). On the other hand, the KN domain of KANK1, which binds talin, displayed similar rapid dynamics at FA in both wild-type and Tpm3 knockout cells (Fig. 6d). Because KANK acts as a bridge that connects FAs to other components of CMSCs, we also examined the possible effects of Tpm3 depletion on ELKS (by antibody staining) and CLASP2 (by expressing mCherry-CLASP2). CLASP2 was enriched in the cell periphery with occasional co-localization with FAs in wild-type cells. Also, ELKS typically localized to the vicinity of FAs. However, in the Tpm3 knockout cells both CLASP2 and ELKS showed predominantly diffuse cytoplasmic localization (Supplementary Fig. 8c, d). Finally, ACF7/MACF1, which is a large scaffolding protein that cross-links microtubule plus-ends and actin filaments at FAs, displayed diminished localization to FAs in the Tpm3 knockout cells (Fig. 7a, b). ACF7/MACF1 displayed somewhat reduced localization to FAs also in the Tpm1 knockout cells. We speculate that the diminished localization of ACF7/MACF1 in Tpm1 knockout may be due to generally smaller FA size in these cells, since ACF7/MACF1 was typically enriched in larger and elongated FAs in wild-type cells (Fig. 7a, b).

To elucidate the molecular interplay between tropomyosin-actin filaments and the above-mentioned proteins involved microtubule – FA interplay, we focused on KANK1 whose C-terminal domain displays similar, yet slightly higher vertical localization in FAs compared to Tpm3.2 (Supplementary Fig. 9d, e), as well as on ACF7/MACF1 and CLASP2, which were previously reported to bind actin filaments^60,61^. Co-sedimentation assays using α-actinin-, Tpm1.6- and Tpm3.2–saturated actin filaments provided evidence that the C-terminal ankyrin-repeat domain of KANK1 does not bind actin, whereas the L-TOG2-S fragment of CLASP2^62^ appears to associate with all three types of actin filaments, representing three actin layers of FAs, with similar affinity in vitro (Supplementary Fig. 10a, b). The N-terminal actin-binding region of ACF7/MACF1, consisting of tandem CH domains^63^, interacted with both Tpm1.6- and Tpm3.2-actin filaments more efficiently as compared to α-actinin cross-linked actin filaments (Fig. 7c).

Together, these results reveal that Tpm3.2 is important for stabilizing ACF7/MACF1, KANK and their interaction partners at adhesions. Hence, Tpm3.2 is critical for proper microtubule – FA interplay, and the loss of Tpm3 consequently leads to defective microtubule-dependent disassembly of FAs.

## Discussion

Our study identifies two new, spatially and functionally distinct actin filament layers in the nanoscale architecture of FAs. These layers are specified by Tpm1.6-decorated actin filaments, which localize slightly beneath the previously described ‘α-actinin layer’, and Tpm3.2-decorated actin filaments, which are positioned towards the bottom of FAs. Importantly, the two tropomyosins have opposite roles in focal adhesion regulation: loss of Tpm1.6 impacts integrin activation, adhesion maturation and cell migration, whereas loss of Tpm3.2 affects adhesion disassembly.

Our findings of Tpm1.6 and Tpm3.2-actin layers being spatially distinct are consistent with previous biochemical studies demonstrating that Tpm1.6 and Tpm3.2 bind actin filaments in a mutually exclusive manner and hence cannot co-exist on the same actin filament. Moreover, both tropomyosins also appear to compete for actin-binding with α-actinin^44,48^, demonstrating that Tpm1.6, Tpm3.2 and α-actinin indeed segregate to distinct actin filament arrays within FAs. Hence, the architecture of FAs is more complex than depicted in current models. This is in accordance with electron microscopy studies highlighting the heterogenous organization of actin filaments within adhesions^64–66^. Our FRAP analysis also provided evidence that FAs are composed of relatively dynamic actin filaments. Moreover, whereas earlier photobleaching and photoactivation experiments demonstrated that actin filaments within dorsal stress fibers undergo retrograde flow^8,51^, the photobleached region at focal adhesion did not ‘treadmill’ from the adhesion to the associated dorsal stress fiber. Thus, actin filaments located at FAs appear relatively short, and they do not predominantly extend to the associated stress fibers (Supplementary Fig. 3f, g). In the future, it will be important to examine how specific actin filament layers are assembled at FAs. We speculate that different actin filament assembly factors in adhesions (Ena/VASP family proteins, APC, and certain formins) may generate specific actin filament layers, and that interactions of these actin filaments with other FA proteins may determine their vertical positioning. In this context, it is interesting to note that VASP, which interacts with actin filament barbed ends to catalyse their polymerization, localizes slightly below the α-actinin layer in the nanoscale strata of FAs^3^. Thus, VASP may be responsible for the assembly of a specific actin filament layer within adhesions. Moreover, in the future it will be important to identify other components associating with the newly identified Tpm1.6- and Tpm3.2-actin layers within FAs, because by forming continuous head-to-tail polymers along the entire length of actin filament different Tpm isoforms regulate interactions of other proteins with these actin filaments to form functionally specific actin filament structures^43^.

Our knockout studies suggest that the two actin filament layers, specified by Tpm1.6 and Tpm3.2, may have different functions in FA dynamics. Tpm1 knockout cells display severe defects in adhesion maturation, and abnormalities in cell migration. These phenotypes were consistent in two different knockout clones, and could be rescued by expression EGFP-Tpm1.6, demonstrating that they indeed arise from the loss of Tpm1.6 (Fig. 3; Supplementary Fig. 6h, i). It is also important to note that, in addition to adhesions, Tpm1.6 localizes to stress fibers, which were thinner and less organized in the Tpm1 knockout cells compared to the wild-type cells (Supplementary Fig. 5 c, d). Furthermore, the retrograde flow of actin in dorsal stress fibers appeared slower and more variable in the Tpm1 knockout cells as compared to the wild-type cells (Supplementary Fig. 5a, b). Thus, we propose that Tpm1.6 contributes to FA maturation by stabilizing the stress fiber network. Alternatively, Tpm1.6-actin filaments may contribute to adhesion maturation by linking FAs to stress fibers. Because intact stress fibers and their proper connection to FAs are critical for actomyosin-based force generation, defects in these functions provide a plausible explanation for the diminished traction forces, and defective force-dependent adhesion maturation in the Tpm1 knockout cells (Fig. 7d). It is also important to note that other splice variants of the *TPM1* gene, Tpm1.8 and Tpm1.9, were earlier shown to contribute to nascent adhesion assembly. However, unlike Tpm1.6, the Tpm1.8 and Tpm1.9 isoforms do not extensively localize to FAs or to stress fibers, but instead form lamellipodial actin filament arrays, which anchor to the nascent adhesions during their assembly^67^. Moreover, our Tpm1 knockout targeted exon 1a of the *TPM1* gene, and thus specifically depleted Tpm1.6 and the closely related Tpm1.7 isoform, without affecting Tpm1.8 or Tpm1.9 (Supplementary Fig. 4a).

Whereas Tpm1.6 contributes to adhesion maturation, the Tpm3.2-actin filaments may be important for regulated adhesion disassembly. Tpm3 knockout cells displayed abnormal accumulation of FAs at the centre and rear (Fig. 7d). Accumulation of unusually stable adhesions also explains the abnormal morphology of the Tpm3 knockout cells, as well as the defects in tail retraction during cell migration. The defects in FA dynamics in Tpm3 knockout cells are unlikely to arise from their abnormal stress fiber network, because although the actin stress fiber phenotypes of Tpm1 and Tpm3 knockout were similar to each other, their effects on focal adhesion dynamics and cell migration were opposite. At the mechanistic level, we revealed that microtubules fail to properly target FAs in Tpm3 knockout cells, and this results in an abnormal organization of the interphase microtubule networks characterized by ‘disorganized’ criss-crossing of microtubules, their elongation past adhesions, and growth in parallel to the cell edge. These phenotypes were consistent in the two knockout clones analysed, and could be partially rescued by expression of EGFP-Tpm3.2 in the knockout cells (Supplementary Fig. 7c, e), demonstrating that these defects indeed result from the loss of Tpm3.2 protein. Interplay between Tpm3.2 and microtubules is also supported by co-immunoprecipitation work with Tpm3.1/2-specific antibody identifying tubulin, EB1, and dynein as proteins associating with Tpm3.1 or Tpm3.2^68^.

How could Tpm3.2-actin filaments contribute to microtubule targeting to adhesions? The diminished resident times of microtubule plus-ends with FAs in the Tpm3 knockout cells correlated with less extensive localizations of ACF7/MACF1, KANK1, KANK2 and ELKS around FAs. These proteins were shown to be critical for targeting microtubule plus-ends to FAs^18^. The precise mechanism by which microtubules promote FA disassembly remains incompletely understood, but at least in some cell-types it involves local inhibition of RhoA^20^. Whether this is also the case in U2OS cells used in our study remains to be shown, but the global levels of active RhoA were decreased, rather than increased in the Tpm3 knockout cells (Supplementary Fig. 4h). In addition to local regulation of RhoA, microtubules contribute to adhesion turnover through delivery of autophagosomes and matrix metalloproteinases to FAs^21,22,69^. Moreover, local acetylation of microtubules by αTAT1, which binds talin at FAs in a mechanosensitive manner, can release GEF-H1 from microtubules to enhance RhoA-mediated stress fiber contractility^70^. Thus, future work is needed to uncover the specific roles of these processes in Tpm3.2/actin/microtubule -dependent FA disassembly.

Although KANK proteins still showed some localization to FAs in the Tpm3 knockout cells, the dynamics of KANK1 were altered by Tpm3 depletion. In wild-type cells, approximately 50 % of GFP-KANK1 was stably associated with FAs, but in the absence of Tpm3 the immobile fraction was lost and the majority of the GFP-KANK1 fluorescence recovered in >30 s. Interestingly, the FA dynamics of the isolated talin-binding KN domain of KANK1, which also lacks a stable fraction in FAs^59^, was not drastically affected in the Tpm3 knockout cells. Therefore, stable association of KANK1 with adhesions appears to require both direct interaction with talin through the KN domain of KANK1, as well as the presence of Tpm3.2-actin filaments (Fig. 7e). In this context, it is also important to note that our iPALM studies revealed similar vertical localizations of the KANK1 C-terminus and Tpm3.2 in FAs. Although we did not find evidence of KANK1 directly binding to Tpm3.2-actin filaments, an earlier study provided evidence that external force is required to stabilize the KANK-talin interaction^59^.Thus, it is tempting to speculate that Tpm3.2-actin filaments associate indirectly with KANK to provide the required force to stabilize KANK-talin interaction at FA. Because ACF7/MACF1 bound tropomyosin-actin filaments more efficiently as compared to α-actinin cross-linked actin filaments, and its localization to FAs was severely diminished in the Tpm3 knockout cells, it is possible that ACF7/MACF1 – tropomyosin-actin interactions have an important role in controlling the localization of KANK and other ‘cortical microtubule stabilizing complex’ proteins at FAs. However, because ACF7/MACF1 binds both Tpm1.6- and Tpm3.2-actin filaments with similar affinities in vitro, other factors, such as actin filament mechanics (please note that Tpm3.2 activates actin-dependent myosin II ATPase, whereas Tpm1.6 does not^44^), and other tropomyosin-binding proteins are likely to contribute to the specific functions of Tpm1.6- and Tpm3.2-actin filaments at FAs. Furthermore, the specific vertical localization of Tpm3.2-actin filament layer towards the bottom of FA may place Tpm3.2-actin filaments in proximity to the ‘cortical microtubule stabilizing complex’ proteins. On the other hand, the Tpm1.6-actin filaments, which are positioned further away from the ‘integrin signalling layer’, may be located too far from the membrane-associated ‘cortical microtubule stabilizing complex’ to properly interact with the actin-binding components of this complex. Nevertheless, further work is required to reveal the specific mechanism by which Tpm3.2-actin filaments associate with ACF7/MACF1, KANK, and other ‘cortical microtubule stabilizing complex’ proteins in cells. Furthermore, elucidating the relationship between the Tpm1.6 and Tpm3.2-actin filaments and the ‘actin regulatory layer’^5^ presents an important challenge for future research.

The opposite functions of Tpm1.6 and Tpm3.2 in FA turnover also provide a plausible explanation for the differential roles of these tropomyosin isoforms in cancer progression. Upregulation of Tpm3.1 and Tpm3.2 is detected in the majority of cancers and transformed cells, and a small molecule inhibitor of Tpm3.1 was shown to kill cancer cells in vitro and in xenotransplants^68,71,72^. On the other hand, Tpm1.6 and Tpm1.7 levels are typically decreased in transformed cells^52,73^. Thus, we propose that over-expression of Tpm3.2, and the closely related Tpm3.1, lead to abnormally dynamic FAs, which allow uncontrolled cell migration and invasion. Similarly, the loss of Tpm1.6 leads to defects in adhesion maturation, and accelerated and uncontrolled cell migration, hence explaining the function of Tpm1.6 as cancer suppressor.

Collectively, our study reveals unexpected complexity in the structural organization of actin at FAs and hence sets a framework for future studies on the nanoscale localization of other FA components and their interplay with the three actin filament layers of adhesions. Our work also suggests that Tpm3.2-actin filaments are important for microtubule – adhesion interactions, and hence for regulated FA turnover. Finally, our study presents an example of three different actin filament populations, potentially with distinct molecular functions, working in concert during the assembly, disassembly and function of a specific actin-based cellular structure.

## Methods

### Reagents and antibodies

The following antibodies were used in this study. Mouse anti-vinculin (Sigma Aldrich, V9131), mouse anti-paxillin (BD Bioscience, 610569), rabbit anti-paxillin (GeneTex, GTX125891), mouse anti-active integrin-β1 (12G10; abcam, ab30394), rabbit anti-NM-IIA (BioLegend, 909801), rabbit anti-NM-IIB (BioLegend, 909901), rabbit anti-vinculin (abcam, 73412), mouse anti-kindlin-2 (Sigma-Aldrich, MAB2617), rabbit anti-SHARPIN (Proteintech, 14626-1-AP), mouse anti-ACF7 (abnova, H00023499-A01) mouse anti-α-tubulin (Sigma Aldrich, T5769), mouse monoclonal anti-EB1 (1A11/4; Santa Cruz, 47704), rabbit anti-KANK1 (Bethyl laboratories, A301-882A), rabbit anti-KANK2 (Sigma-Aldrich, HPA015643), mouse anti-talin (Sigma Aldrich, T3287), tubulin, rabbit anti-ERC1 (ELKS) (Sigma-Aldrich, HPA019513), rabbit anti-Y118-phosphorylated paxillin (Cell Signaling Technology, 2541), antibody against Tropomyosin1, 2; mouse anti-Tropomyosin 1 and 2 (T2780, clone name-TM311, Sigma-Aldrich, 014M4782) and mouse anti- Tropomyosin 3 (CG3). 4’,6’-diamidino-2-phenylindole (DAPI) (Thermo Fisher Scientific, D1306) was utilized to detect the nuclei, whereas Alexa Fluor 488- and 568-Phalloidin (Thermo Fisher Scientific, A12379/12380) were applied to visualize F-actin. Alexa Fluor 488- and 568-conjugated goat anti-mouse (Thermo Fisher Scientific, A-11001 and A-11031, respectively) and Alexa Fluor Plus 647-conjugated goat anti-rabbit (Thermo Fisher Scientific, A32733) were used as secondary antibodies. 5% BSA-TBS-Tween20 (0.02%) was used as a blocking buffer for the cells prior to staining as well as a diluent for the antibodies mentioned above. Polyclonal rabbit ab against GAPDH (Sigma-Aldrich, G9545) was used to probe equal loading in WB. Fibronectin (10 µg/ml, 11080938001, Merck) was used for surface-coating in live and fixed-cell imaging studies. Treated coverglass was mounted using ProLong™ Glass Antifade Mountant (P36980, Invitrogen). Plasmids used in this study are listed in Supplementary Tables 2 and 3.

### Cell culture and transfection

Human osteosarcoma (U2OS) cells (authenticated by ECACC through STR-profiling method to be the same origin as the original U2OS cell line; case number-13472) were maintained as previously described in refs. ^42,74,75^. Briefly, cells were cultured in 4.5 g/L glucose containing DMEM (BE12-614F, LONZA), supplemented with 10% fetal bovine serum (10500-064, GIBCO) and Pen-Strep-Glutamine solution (10378016, GIBCO) in a humidified atmosphere at 37 °C, 5% CO_2_ and 95% relative humidity. Cells were regularly tested for mycoplasma contamination using the Mycoalert™ Mycoplasma Detection Kit (LT07-418, LONZA). For live-cell imaging experiments, Fluorobrite DMEM (Gibco, A1896701), supplemented with 25 mM HEPES, glutamax and 10% FBS was used. Transient transfections were performed either with Xfect transfection reagent (Takara, 631318) or with Mirus Bio TransIT-2020 transfection reagent (MIR 5400) according to the manufacturers’ instructions. Transfections were carried out overnight before live-cell imaging or before fixation. For rescue experiments, cells were transfected for 24 h. After transfection, cells were either fixed with 4% PFA in PBS for 15 min at room temperature or detached with 0.05% Trypsin-EDTA (Gibco, 15400054) and plated onto high-precision (#1.5H) 35 mm imaging dishes (Ibidi µ-dish high, 81158) or #1.5H coverslips coated with 10 µg/ml fibronectin. For live-cell imaging, cells were allowed to adhere for three hours prior to placing them in the imaging chamber. For live-cell imaging on micropatterns, wild-type U2OS cells were transfected with EGFP-Tpm1.6 and EGFP-Tpm3.2 along with miRFP670-paxillin for 24 h, and re-plated before imaging for 2 h on fibronectin pre-coated CYTOOchips™ crossbow micropatterns 35 mm coverglasses (10-600-10-18, Cytoo) attached with a CYTOOchamber™ (30-010, Cytoo).

### Generation of Tropomyosin 1 and Tropomyosin 3 knockout cell lines

Tropomyosin 1 and Tropomyosin 3 CRISPR knock-out cell lines were generated as described previously^42,74,76^. Guide sequences targeting exon 1a (CTCGACAAGGAGAACGCCT) of the human *TPM1* gene and exon 1b (GAGAAGTTGAGGGAGAAAGG) of *TPM3* gene were selected based on the CRISPR Design tool with highest on target efficiency scores. Oligonucleotides for cloning guide RNA into pSpCas9 (BB)-2A-GFP vector (48138; a gift from F. Zhang, Addgene, Cambridge, MA) were designed as described previously^77^. Transfected cells were sorted with FACSAria II (BD), using low intensity GFP-positive pass gating, as single cells onto a 96-well plate, supplemented with DMEM containing 20% FBS and 10 mM HEPES buffer. For this study, two CRISPR clones were selected for each KO based on the lack of detectable Tpm1 and Tpm3 proteins by Western blot. All the data presented in the manuscript for Tpm1 and for Tpm3 knockouts are from clones 1 and 2, respectively. Data for Tpm1 knockout clone 2 and for Tpm3 knockout clone 11 are shown in Fig. 3. For further validation of tropomyosin knockouts we performed Sanger Sequencing (Eurofins genomics sequencing service) and next generation sequencing (NGS) (Illumina MiSeq.). For Tpm1 knockout clone 1, CRISPR resulted in deletion of 17 nucleotides that eventually leads to a frameshift and appearance of STOP codon soon after the deletion site. The results were confirmed by NGS (MiSeq, Illumina). For Tpm3 knockout clone 2, CRISPR resulted in insertion of a single nucleotide (A) that eventually leads to a frameshift and appearance of STOP codon soon after the deletion site. NGS was performed at the DNA Sequencing and Genomics Laboratory (BIDGEN) laboratory (Institute of Biotechnology, University of Helsinki, Finland).

### Generation of EGFP-CAAX cell line

The pEGFP-CAAX plasmid was made by adding the CAAX-motif (encoding the peptide CMSCKCVLS) of H-Ras to the C-terminus of EGFP by using inverse PCR. EGFP-CAAX was then cloned into the safe harbor vector pSH-FIRE-P-AtAFB2 (Addgene, #129715) that was integrated into the safe harbor of U2OS cells using CRISPR/Cas9. Stable cells expressing EGFP-CAAX were obtained by puromycin selection.

### Cloning of GFP-KN-L1-KANK1

All plasmids were made by Gibson assembly of PCR products. The GFP-KN-L1-KANK1 was made by PCR products of GFP vector and KANK1-KN-L1 insert from GFP-KANK1 construct (pPL1866). PCR products were cloned according to primers listed in Supplementary Table 2, and Gibson assembly was made with KAPA HiFi (ReadyMix x2 #KK2601) according to manufactures instructions.

### Immunofluorescence staining

Cells were cultured on 10 µg/mL fibronectin-coated (Sigma-Aldrich, L2020) coverslips or on 35 mm imaging dishes (Ibidi µ-dish high). Except for microtubule and EB1 staining, cells were fixed with 4% PFA in PBS at RT for 15 min, washed several times with PBS, permeabilized with 0.2% Triton X-100 in PBS at RT for 7 min and washed again with PBS. Cells were blocked with 5% BSA in PBS for 1 h at RT and incubated with primary antibodies in 5% BSA for 1 h at room temperature. Cells were washed several times with PBS-T (0.02% Tween-20 in 1X PBS), and incubated with secondary antibodies for 1 h at RT. After several washing with PBS-T, coverslips were mounted onto microscopes slides with ProLong™ Glass Antifade Mountant (P36980, Invitrogen). Microtubule staining was performed as described before^78^. In brief, cells were fixed with 3% PFA + 0.025% Glutaraldehyde in Cytoskeletal Buffer (CB) supplemented with 10% Sucrose (Sucrose was added to the buffer just before the use) stock solution containing 10 mM HEPES (H3375, Sigma) at pH 6.1, 138 mM KCl (P3911, Sigma), 3 mM MgCl2 (208337, Sigma) and 2 mM EGTA (E3889, Sigma) at room temperature for 10 min. Cells were washed with PBS and incubated with a solution of 1 mg/mL sodium borohydride in PBS for 10 min at RT. Cells were then permeabilized with 0.2% Triton X-100 in PBS at RT for 7 min. For EB1 staining, cells were fixed with cold methanol for 5 min on ice. For staining on micropatterns, cells were plated on fibronectin pre-coated CYTOOchips™ crossbow micropatterns 35 mm coverglasses (10-600-10-18, Cytoo) and were allowed to spread for 2 h before fixation. For 12G10 staining on micropatterns, 37 µm crossbow micropatterns were generated as previously described in ref. ^79^ and coated with 10 µg/mL fibronectin and 5 µg/mL Alexa Fluor^TM^ 647-conjugated BSA. Cells were seeded for 3 h prior to being fixed and stained.

### Western blotting

Cell lysates were prepared by washing the cells once with cold PBS and scraping them into lysis buffer (50 mM Tris-HCl pH 7.5 150 mM NaCl, 1 mM EDTA, 10% Glycerol, 1% Triton X-100) supplemented with 1 mM PMSF, 10 mM DTT, 40 mg/ml DNase I and 1 mg/ml of leupeptin, pepstatin, and aprotinin. All preparations were conducted on ice. Protein concentrations were determined with Bradford reagent (#500-0006, Bio-Rad) and equal amounts of the total cell lysates were mixed with Laemmli Sample Buffer, boiled, and run on 4%–20% gradient SDS-PAGE gels (#4561096, Bio-Rad). Proteins were transferred to nitrocellulose membranes with Trans-Blot Turbo transfer system (Bio-Rad) using Mini TGX gel transfer protocol. Membranes were blocked in either 5% milk-TBS with 0.1% Tween20 (TBS-T) or with 5% BSA for one hour at RT. Primary and secondary antibodies were diluted into fresh blocking buffer for overnight incubation at 4 °C and one hour at room temperature, respectively. Protein detection from the membranes was performed with Western Lightning ECL Pro substrate (PerkinElmer NEL121001EA).

### Protein purification

The plasmid encoding pHIS9-MBP-ACF7 residues 73-306 fragment (plasmid library no. pPL2015) with the Y259D activating mutation^63^, was introduced into *E. coli* BL21(DE3) cells to express the protein. A 500 ml culture was inoculated with a 5 ml starter culture and grown in auto-induction medium (AIMLB0210, Formedium) for 24 h at RT (at 21 °C). The medium was supplemented with kanamycin antibiotic and antifoam 204 (A6426, Sigma) while shaking at 220 rpm in a 2 L flat-bottom glass Erlenmeyer flask. Subsequently, the cells were pelleted, resuspended in 35 ml of Ni-Binding Buffer (50 Tris pH 7.5, 300 mM NaCl, 10 mM Imidazole, 0.02% NaN3) in a 50 ml falcon tube, flash-frozen in liquid N_2_, and stored at −80 °C until further use. Upon thawing in RT water and transferring to ice, protease inhibitors (200 µg/ml PMSF, 1 µg/ml leupeptin, 1 µg/ml aprotinin, 1 µg/ml pepstatin A, 1 mM benzamidine), along with 50 µg/ml lysozyme and 20 µg/ml DNAseI, were added. The cells were sonicated for 1 min at 70% power, 50% cycle, 4 times (Sonopuls, Bandelin, Germany). The resulting cell lysate was clarified by centrifugation for 1 h at 48,000 x g at +4 °C. The supernatant after centrifugation was loaded onto a 5 ml HisTrap FF Ni-NTA column (Cytiva, #17525501) connected to an AKTA pure FPLC instrument. The column was thoroughly washed with Ni-Binding buffer, and the protein was eluted by applying 250 mM Imidazole in Ni-Binding Buffer using reverse flow to minimize the elution volume with a high protein concentration. The eluted protein (approximately 4 ml) was immediately subjected to gel filtration chromatography (without protein concentration) using a HiLoad 16/600 Superdex 75 column at a flow rate of 1 ml/min. The column was pre-equilibrated with GF-buffer (20 mM HEPES, 50 mM NaCl, 0.03% NaN3). A single symmetric peak containing the protein was collected and concentrated in a 10k MWCO Amicon Ultra concentrator (UFC801096, Millipore) up to 88 µM. The protein concentration was determined using a nanodrop, with an Abs 0.1% ( = 1 g/l) reading of 1.361 (calculated using protparam software). The concentrated protein was aliquoted, flash-frozen in liquid nitrogen, and stored at −80 °C for further experiments.

The GFP-L-TOG2-S fragment of human CLASP2 (residues 261-793) (plasmid library no. pPL2014) was expressed and purified similarly to the ACF7 protein, with minor modifications in buffer compositions. The Ni-NTA Binding Buffer consisted of 50 Tris pH 7.5, 300 mM NaCl, 10 mM Imidazole, 5% glycerol, 1 mM DTT, and 0.02% NaN3. The Ni-NTA Elution Buffer contained 50 Tris pH 7.5, 300 mM NaCl, 250 mM Imidazole, 5% glycerol, 1 mM DTT, and 0.02% NaN3. The GF-buffer used was composed of 50 mM HEPES pH 8.0, 500 mM NaCl, 5% Glycerol, 1 mM DTT, and 0.02% NaN3. The gel filtration profile exhibited several peaks, with the largest peak being GFP-positive, which was selected, concentrated, and flash-frozen like the ACF7 protein.

The plasmid encoding the His9-SUMO-terminal ankyrin repeat domain fragment of human KANK1 (residues 1073-1353) (plasmid library no. pPL2017), was introduced into *E. coli* BL21-CodonPlus (DE3)-RIPL cells (Agilent Technologies, # 230246-3) for protein expression. A 500 ml culture was grown in AIM (AIMLB0210, Formedium) for 24 h at RT (at 21 °C), supplemented with kanamycin antibiotic and antifoam 204 (A6426, Sigma). This culture was agitated at 220 rpm in a 2 L glass Erlenmeyer flask with a flat bottom. The cells were then pelleted, resuspended in 35 ml of Ni-Binding Buffer (50 Tris pH 7.5, 300 mM NaCl, 10 mM Imidazole, 1 mM DTT, 1 mM EDTA, 5% glycerol, 0.02% NaN3) in a 50 ml Falcon tube, flash-frozen in liq. N_2_, and stored at −80 °C until further use. Upon purification, the cells were thawed in RT water and transferred to ice once liquid. Protease inhibitors (200 µg/ml PMSF, 1 µg/ml leupeptin, 1 µg/ml aprotinin, 1 µg/ml pepstatin A, 1 mM benzamidine), 50 µg/ml lysozyme, and 20 µg/ml DNAseI were added, and the cells were sonicated for 1 min at 70% power, 50% cycle, 4 times (Sonopuls, Bandelin, Germany). The cell lysate was clarified by centrifugation for 1 h at 48,000 x g at 4 °C. The supernatant after centrifugation was applied to a 5 ml HisTrap FF Ni-NTA column (Cytiva, #17525501) connected to an AKTA pure FPLC instrument. The column was extensively washed with Ni-Binding buffer, and the protein was eluted by applying 250 mM Imidazole in Ni-Binding Buffer. The eluted protein was immediately mixed with SENP2 protease to cleave off the SUMO tag and subjected to dialysis against Buffer A (10 Tris pH 7.5, 10 mM NaCl, 1 mM DTT, 1 mM EDTA, 2.5% glycerol) for 2 h at +4 °C. Then the protein was centrifuged to remove the aggregated protein fraction and diluted approximately 10-fold with Buffer A. It was immediately applied to an anion exchange HiTrap Q FF column (Cytiva, #17505301) and eluted with a salt gradient using Buffer A + 1 M NaCl on an AKTA Pure FPLC. The protein peak eluted in the middle of the gradient with around 450–500 mM NaCl and 20 mS/cm conductivity. The fractions containing protein were collected, pooled together, and the concentration was measured to be around 21.4 µM. The protein was aliquoted, flash-frozen with liquid nitrogen, and stored at −80 °C for further experiments.

The plasmid encoding human His6-α-actinin-4 was expressed in *E. coli* BL21(DE3) cells and purified using the Ni-NTA affinity method, similar to pHIS9-MBP-ACF7(73-306). The fractions containing pure His6-α-actinin-4 protein, as observed from Coomassie-stained SDS-PAGE gels, were pooled together, concentrated, diluted 10x with buffer A (20 mM HEPES at pH 7.5, 5 mM NaCl, and 0.02% NaN_3_), and loaded to an anion-exchange HiTrap Q FF column (Cytiva, #17505301). The protein was eluted using salt gradient with buffer A + 500 mM NaCl, and fractions containing protein were pooled and subjected to gel filtration chromatography using a HiLoad 16/600 Superdex 75 column with a flow rate of 1 ml/min. This column was pre-equilibrated with the GF-buffer (20 mM HEPES, 50 mM NaCl, 0.03% NaN_3_). The fractions containing pure His6-α-actinin-4 were concentrated and flash-frozen, similar to the ACF7 protein.

### Actin filament binding assay

Actin filament binding experiments were conducted following the procedures outlined in^74^, with some minor adjustments. Non-muscle actin (specifically, β/γ-actin from human platelets) was acquired from Cytoskeleton Inc. and utilized according to the manufacturer’s guidelines. Varying quantities of either β/γ-actin or a combination of β/γ-actin, α-actinin-4, and non-tagged Tpm1.6, or Tpm3.2 were mixed in the presence of G-buffer (comprising 5 mM HEPES at pH 7.4, 0.2 mM CaCl2, 0.2 mM DTT, and 0.2 mM ATP). To ensure tropomyosin saturation on actin, we maintained a 4:1 ratio of actin to tropomyosins. However, to prevent actin filament sedimentation due to cross-linking with α-actinin-4, α-actinin-4 was directly added to the reaction tubes in a 1:1 ratio with actin. The concentrations of KANK1 ankyrin repeats, GFP-L-TOG2-S (CLASP2a), and ACF7 Y259D were consistently maintained at 1 μM for all experiments, and all reactions were conducted at room temperature. Polymerization was initiated and carried out for 30 min using F-buffer (comprising 20 mM HEPES at pH 7.4, 100 mM KCl, 5 mM MgCl2, 0.2 mM EGTA, 1 mM DTT, and 2 mM ATP). Following polymerization, 1 μM of KANK1 ankyrin repeats, GFP-L-TOG2-S (CLASP2a), or ACF7 Y259D were added to the polymerized actin, or actin in combination with tropomyosin or actin with α-actinin-4, and the mixture was incubated for an additional 30 min. The ACF7 Y259D complex was subjected to centrifugation at 436000 x g for 60 min at 10 °C, while the KANK1 and GFP-L-TOG2-S (CLASP2a) complex were centrifuged at 109,000 x g for 30 min at 10 °C using a TLA100 rotor in a Beckman Optima MAX Ultracentrifuge. Supernatant and pellet fractions were prepared for SDS–PAGE analysis by adding Laemmli buffer. Protein bands were separated on 4–20% gradient SDS–PAGE gels (Mini-PROTEAN TGX Precast Gels, Bio-Rad Laboratories Inc.) and stained using a 1% Coomassie staining solution (consisting of 25% isopropanol and 10% glacial acetic acid with Coomassie brilliant blue R-250, 1610400, Biorad Laboratories, Inc.). The intensities of KANK1 ankyrin repeats, GFP-L-TOG2-S (CLASP2a), and ACF7 Y259D bands were quantified using the QuantityOne program (Bio-Rad). The ACF7 Y259D co-sedimentation assay data were plotted, with the concentration of ACF7 Y259D in the pellet (in μM) on the Y-axis and the concentration of actin, or actin in combination with α-actinin-4 or tropomyosins (in μM) on the X-axis.

### MICROSCOPY

#### Widefield

Widefield imaging for IF samples were performed with Leica DM6000B wide-field fluorescence microscope equipped with a 63x/1.40-0.60 HCX PL APO Lbd.bl. Oil wd=0.10 and 40x/1.25-0.75 HCX PL APO Oil wd=0.10. The images were acquired using Hamamatsu Orca-Flash4.0 V2 sCMOS camera with image resolution 2048 × 2048 pixels.

### TIRF

Fixed cell imaging on 35 mm imaging dishes (Ibidi µ-dish high) in 1× PBS was performed with Ring-TIRF module of Deltavision OMX SR (Cytiva) with 60×/1.49NA Apo N oil objective (Olympus), at RT, using 1.518 RI immersion oil and 488, 561 and 607 nm diode lasers. 5 × 5 FOVs (1024 × 1024) including 10% overlap, were captured manually, followed by moving to a new area, at least over six times the FOV to another direction, at random positions on the imaging dish. Live cell imaging was performed as fixed cell TIRF experiments, but with following exceptions: imaging was performed with 1.522 RI immersion oil and imaging chamber with controlled humidified atmosphere of 5% CO_2_ and 37 °C was utilized. Sample illumination with 488, 561, and 607 nm diode lasers was detected and recorded with three respective sCMOS cameras and controlled through Acquire SR 4.4 acquisition software. The captured 1024 × 1024 time-lapse videos had a pixel size of 0.08 µm (x/y). The time-lapse imaging for miRFP670-paxillin expressing cells was performed with 30 s interval for a total of 2 h. 607 laser was used at 25% laser power with 100 ms exposure time. The time-lapse imaging for cells co-expressing miRFP670-paxillin either with GFP-α-tubulin or EGFP-EB1 was performed with 3 s and 1 s intervals for a total of 5 min and 1 min, respectively. 488 and 607 lasers were used at 15 % laser power with 50 ms exposure time. Obtained time-lapse series were deconvolved and channels aligned with SoftWoRx 7.0. Prior the onset of live-cell imaging, cells were allowed to settle within the imaging chamber for 30 min. FRAP experiments were also performed with Deltavision OMX SR. Live-cell imaging for tropomyosin recruitment to adhesions was performed with TIRF module of ONI Nanoimager S with 100x/1.49 NA oil objective at 37 °C on 35 mm imaging dishes (Ibidi µ-dish high). Sample illumination with 488, 561, 640 nm lasers was detected and recorded with sCmos camera with field of view of 50 µm x 80 µm. 488, 561 and 640 lasers were used at 3% laser power with a 400 ms exposure time. The time-lapse imaging for miRFP670-Paxillin, EGFP-Tpm1.6, mRuby2-Tpm3.2 expressing cells was performed with 30 s interval for 1.5 h.

### iPALM

iPALM imaging was performed similar to as previously described in refs. ^3,30^ on U2OS cells plated on 25 mm diameter round coverslips containing gold nanorod fiducial markers (Nanopartz, Inc.), passivated with a ca. 50 nm layer of SiO2, deposited using a Denton Explorer vacuum evaporator, except the coverslips were not coated with any ECM protein for plating the cells. Cells were plated for 18 h before fixation. After fixation an 18 mm coverslip was adhered to the top of the sample and placed in the iPALM. Eos-tagged samples were excited using 561 nm laser (Opto Engine LLC) excitation at ca. 1–2 kW/cm^2^ intensity in TIRF conditions. Photo- conversion of Eos was performed using 405 nm laser (Vortran Laser Technology Inc.) illumination at 2–10 W/cm^2^ intensity. 50,000–80,000 images were collected through dual Nikon Apo TIRF 60x/1.49NA objective lenses coupled to a 593/40 nm bandpass filter (Semrock), and acquired via three EMCCD cameras (DU987U, Andor) 50 ms exposure, and processed/ localized using the PeakSelector software (Janelia Research Campus^80^; Alexa Fluor 647-labelled paxillin was imaged similarly, but with 2–3 kW/cm^2^ intensity 647 nm laser excitation (Opto Engine, LLC.), 647 nm long-pass filter (Semrock), and 30–40 ms exposure time in STORM-buffer containing TRIS- buffered glucose, glucose oxidase, catalase, and mercaptoethanol amine^30,80^. iPALM data were analysed using iPALM plotter (AIC, Janelia Research Campus; https://github.com/aicjanelia/ipalmplotter) and images were rendered using the PeakSelector software (Janelia Research Campus). iPALM localization data records both the fluorescent molecules localized within the FA, as well as molecules in the cytoplasmic fraction. To quantify the spatial distribution of the proteins within individual FA, we created FA mask based on the paxillin image by using MATLAB code provided by Janelia AIC. Moreover, the gold nanorod fiducial markers were removed from the rendered images by MATLAB code provided by Janelia AIC. To render iPALM images, a single color scheme was used from red to purple, covering the z range 0–250 nm, where features within FA are seen. The same color scheme was also used for side-view (xz) images and for covering the z range 0–500 nm. For analysis of protein distributions in FA:Zcentre calculation, the three-dimensional molecular coordinates for each region (individual FA) were analysed to obtain histograms of vertical positions with 1-nm bins. The center vertical positions (Zcentre) was determined from a Gaussian fit to the FA molecule peak. For proteins like and α-actinin, where dual peaks were observed, the fitting was done using the sum of two-Gaussian distributions with independent center vertical position and width. After the histograms for all images and individual FA were obtained, they were combined into a single average Zcentre.

### Fluorescence recovery after photo bleaching (FRAP) and photoactivation experiments

The FRAP experiments were performed using a Delta Vision OMX SR microscope with a 1.49 Oil ApoN x60 TIRF objective. Acquisition was performed using AcquireSR (Cytiva). A488 and A607 lasers were used at 25% and 35% laser power, respectively, with a 50 ms exposure time. Imaging was performed with 2 s time-lapse intervals for the total duration of 5 min, except for EGFP-actin, where imaging was performed with 5 s time-lapse intervals for the total duration of 10 min. GFP-KANK1, GFP-KN-L1-KANK1, EGFP-Tpm1.6, or EGFP-Tpm3.2, and EGFP-actin at FAs were bleached with single 0.05 s pulse of A488 laser at 25% laser power. Photoactivation experiments were performed with Deltavision OMX SR as previously described in ref. ^75^. Briefly, 4% laser power (405 nm) in EPI mode with 1 ms exposure was applied to activate PA-GFP-Actin on a dorsal stress fiber visualized by mKate1.31-LifeAct adjacent to a miRFP670-paxillin positive adhesion. The time-lapse imaging with 10 s interval for a total of 5 min was conducted in Highly Inclined and Laminated Optical sheet (HILO) mode by recording also three frames prior to photoactivation. The channels for the obtained time-lapse series were aligned with SoftWoRx 7.0. Prior the onset of live-cell imaging, cells were allowed to settle within the imaging chamber for 1 hr.

### Confocal

Confocal imaging was performed using a 3i Marianas CSU-W1 spinning disk confocal microscope. Fixed cell imaging was performed using a 100x Zeiss Plan-Apochromat 1.4 NA objective, with sample illumination with 405, 488, 561 and 640 nm lasers detected by a Hamamatsu sCMOS Orca Flash4.0 camera (2048 × 2048 pixels). For TFM, imaging was performed at 37 °C using a 40x Zeiss LD C-Apochromat 1.1 NA aperture, and sample illumination with 405, 488, 561 and 640 nm lasers recorded by a Photometrics Evolve 10 mHz Back illuminated EMCCD camera (512 × 512 pixels). Due to the bright intensity of the beads used for TFM, the 561 nm laser was used at 10 % power.

### Traction force microscopy

#### TFM gel preparation and surface activation

First, 35 mm glass bottom dishes (D35-14-1, Cellvis) were incubated with 1 ml bind silane solution (7.14% Plus One Bind silane (GE17-1330-01, Sigma), 7.14% acetic acid in absolute ethanol) for 1 h at room temperature, before being washed twice with 2 ml absolute ethanol and let to air-dry. To prepare 9.6 kPa hydrogels, 1.7 µl of sonicated (30 s on, 30 s off for 7 min) fluorescent beads (FluoSpheres^TM^ 200 nm red, F8810, Life Technologies) were added to a 500 µl hydrogel mixture containing 94 µl 40% acrylamide (A4058, Sigma), 50 µl *N,N’*-methylenebisacrylamide solution (M1533, Sigma) in PBS and briefly vortexed. Hydrogel polymerization was induced through addition of 5 µl 10% ammonium persulfate (1610700, Bio-Rad) and 1 µl *N,N,N’N’*-tetramethylethylenediamine (T9281, Sigma), and the mixture was rapidly vortexed before 11.8 µl was added to the prepared dishes and a clean 13 mm glass coverslip placed on top. Hydrogels were allowed to polymerize for 1 h at RT, before PBS was added and the coverslip was removed. To activate the hydrogel surface, gels were incubated with 500 µl 0.2 mg/ml Sulfo-SANPAH (803332, Sigma), 2 mg/ml *N*-(3-Dimethylaminopropyl)-*N*′-ethylcarbodiimide hydrochloride (03450, Sigma) in 50 mM HEPES for 30 min at RT with gentle agitation before being irradiated with UV light for 10 min. Gels were washed four times with sterile PBS before being coated with 10 µg/ml fibronectin at 4 °C overnight.

### Traction force microscopy

Cells were plated onto the 9.6 kPa gels for 4 h prior to imaging. One hour prior to imaging, the cell media was replaced with media containing 50 mM HEPES (H0887, Sigma), 60 pM SiR-Actin (SC001, Spirochrome), 5 µg/ml Hoechst 33342 (H3570, Invitrogen) to allow visualisation of cells. To enable detection of traction forces exerted by the cells, beads were imaged before and after cell removal (using 20 µl pre-warmed 20% SDS in milli-Q H_2_O). To correct for drift, pre- and post-cell removal bead images were aligned using the NanoJ-Core plugin^81^. Bead tracking and force measurements were performed in MATLAB (Mathworks, version 2020a) using TFM software^82^. For displacement field calculation, high resolution subsampling of beads was used, with no outward deformation expected, subpixel correlation via image interpolation selected, and a template size of 21 pixels with a maximum displacement of 20 pixels. For displacement field correction, vector outliers were filtered and a threshold for the normalized displacement residual of 2. Force field calculation was performed using Fourier transform traction cytometry (FTTC) with a regularization parameter of 0.0001. Actin cell masks were generated from sirAct images in Fiji/ImageJ and overlaid onto traction maps in R (R Core Team (2022). R: A language and environment for statistical computing. R Foundation for Statistical Computing, Vienna, Austria. https://www.R-project.org/.) to obtain mean traction (Pa) per cell.

### Random cell migration assay

Phase contrast time-lapse imaging of migrating cells was conducted in continuous cell culturing platform Cell-IQ (CM Technologies). Twelve-well plates (Greiner) were coated with 10 µl/ml of fibronectin and cells were allowed to adhere for 2 h prior to imaging. Cells were once washed with PBS and replaced with DMEM containing 10 mM HEPES prior to starting the imaging. The plate lid was switched to Cell-Secure (CM Technologies) enabling insertion of CO_2_ input and output valves. 5% CO_2_-flow was set cycling between 8 min on, 20 min off. Average migration velocity of wild-type, Tpm1 and Tpm3 knockout cells was quantified by tracking the nucleus movement in between 8 min imaging cycles for 25 h with Cell-IQ analyzer (CM Technologies). The cells that did not collide with one another were selected for the analysis.

### RhoA activity assay

The active RhoA was determined by using G protein-linked (G-Lisa) assay (Cytoskeleton, BK124) as described before in ref. ^83^. Briefly, cells were washed on ice with cold PBS and homogenized to ice-cold lysis buffer. Protein concentration was measured and adjusted to 0.9 mg/ml, and samples were snap-frozen with liquid nitrogen. Triplicate assays were done, active RhoA was measured according to the manufacturer’s instructions with absorbance of 490 nm.

### Image analysis

#### Cell morphology analysis

Cells were plated on fibronectin-coated coverslips, fixed after 90 min and 8 h post-plating and stained for actin (phalloidin) and nucleus (DAPI). The images were acquired either with Floid wide-field microscope equipped with Plan Fluorite 20x/0,45 objective and Sony 1.3MP 1/3” ICX445 EXview HAD CCD camera using phase transmitted light channel or with Leica DM6000B wide-field fluorescence microscope. The cell circularity and aspect ratio were quantified by using FIJI/ImageJ. Cell boundaries were drawn manually by using the free hand tool and the measurements taken using the ROI manager tool.

### Quantification of focal adhesion density

The FA density per cell on tissue culture plates was calculated according to the equation described in ref. ^70^:

FA density = Number of FAs in the region (cell) / Area of the region (cell).

Quantification of focal adhesions on micropatterns was performed by using Fiji/ImageJ. A binary FA mask was created by using paxillin channel for images taken at TIRF plane, and the total numbers of FAs and their average sizes per cell were analyzed by wand tracing tool with ROI manager.

### Quantification of Tpm recruitment to focal adhesions

The dynamics of Tpm recruitment to FAs were measured using Fiji/imageJ. Visual analysis of movies identified the region and the frame where a FA started to form (paxillin signal appeared). Using a custom macro, the fluorescence signal from the all channels were measured for 10 frames before and 20 frames after the FA appearance.

### Quantification of focal adhesion spatial distribution

The FA percentage was analyzed in two regions of cells. Cell edge was defined by distance of 5 µm taken from cell periphery and rest of the region as cell center/rear. The percentage of adhesions in these regions was correlated to the total number of adhesions in the same cell.

### Analysis of focal adhesion size distribution

FA areas were quantified with Fiji/ImageJ, measuring the size of each individual adhesion with the ROI manager and freehand tool. The images were processed with the rolling ball background subtraction using 50-pixel ball radius in Fiji/ImageJ, converted to binary, and each individual adhesion analyzed with the ‘analyze particle tool’ from the adhesions’ mask created from binary images. The size threshold was set from the range of 0.08–15 µm^2^. The value 0.08 µm^2^ was selected because our vinculin antibody visualized also unspecific background particles below the size of 0.08 µm^2^. Cells that were in contact with neighboring cells were discarded from the analysis. Adhesions were classified into four groups based on size, and the percentage ratio of the FAs in each group was obtained by dividing the FA number of individual size groups with the total number of focal adhesions in the cell.

### Quantification of focal adhesion properties

Cells transfected with miRFP670-Paxillin were allowed to adhere on fibronectin coated glass-bottom dishes (Ibidi µ-dish high) for 3 h prior to imaging. Time-lapse images were acquired in the interval of 30 sec for the duration of 1.5 h at TIRF plane using of Deltavision OMX SR (Cytiva) with 60×/1.49NA Apo N oil objective (Olympus). The time-lapse movies were stabilized by using the ‘image stabilizer’ plugin in Fiji/ImageJ. The movies were further processed using the Focal adhesion analysis server (https://faas.bme.unc.edu);^56,84^ to analyze the lifetime, assembly, and disassembly rates of all paxillin-positive adhesions per cell.

### Quantifications of KANK2 and ACF7/MACF1 in focal adhesions

The intensity measurements for endogenous KANK2 and ACF7/MACF1 were performed on images taken on TIRFM. First, FA masks were prepared by using channels with either talin or vinculin staining. The images were processed with the rolling ball background subtraction using 50-pixel ball radius in Fiji/ImageJ, converted to binary, and each individual adhesion analyzed with the ‘analyze particle tool’ from the FA mask created from binary with Fiji/ImageJ, using the ROI manager tool. In the case of KANK2 intensity analysis, mean intensity was measured for adhesion size starting from 0.5 µm^2^. Since ACF7 intensity signal was mainly diminished from adhesion smaller than 1 µm^2^ in wild-type U2OS cells, ACF7 mean intensity was measured for adhesion size starting from 1 µm^2^. The background mean intensity was measured from the cytoplasmic intensity of KANK2 and ACF7 staining and were subtracted from their mean intensity measurements at the focal adhesions.

### Stress fiber analysis

The stress fiber analysis was performed by using Ridge detection plugin from Fiji imageJ as described before in ref. ^42^. The parameters used for quantifying the total number of stress fibers were: line width 20.0, high contrast 230, low contrast 100, sigma 6.57, low threshold 0.0, and upper threshold 0.34. The parameters used for quantifying the thick bundles were: line width 29.0, high contrast 230, low contrast 87, sigma 8.87, low threshold 0.0, and upper threshold 0.17.

### 12G10 focal adhesion analysis

Cells were plated onto fibronectin-coated polymer dishes (Ibidi, 8 well µ-slide polymer) for 3 h before being fixed and stained for active integrin-β1 (12G10), paxillin, actin (phalloidin), and DAPI. Images were acquired using a spinning disc confocal microscope. Cell masks were generated from actin staining, and active integrin-β1 intensity calculated from the mean integrated density of the cell mask. Quantification of active integrin-β1 FAs was performed in Fiji/ImageJ using an analysis pipeline adjusted from Horzum, Ozdil and Pesen-Okvur, 2014. 12G10 images were processed using subtract background with a rolling ball radius of 20, the local contrast enhanced using the CLAHE plugin (block size = 19, histogram = 256, maximum slope = 5, no mask) and the background minimised further using EXP before thresholding to generate a binary mask. An adjustable watershed was applied to aid the detection of individual adhesions, and adhesions were analysed using the Analyze Particles tool, with a size threshold set to 0.1 to 15 µm^2^. For active integrin-β1 intensity on micropatterns, cells were plated onto micropatterns for three hours before being fixed and stained for active integrin-β1 (12G10). The micropatterns were used to align cells and mean intensity maps generated by overlaying cells and averaging the intensity. Quantification of 12G10 intensity was calculated from the mean integrated density of a standardised kite-shaped mask (generated by connecting the arms of the cross-bow to the tail).

### FRAP analysis

FRAP data in all Figurs were analyzed similar to the previously described approach^74,76,85^. In brief, first the background subtraction and correction of fluorescence bleaching during imaging (the fluorescence within the region of interest was divided by that of an identical-sized region within the cell) was performed with the help of Fiji/ImageJ. Recovery rate was normalized to the prebleach intensity in every experiment. Data were analysed using Microsoft Excel.

### Quantification of microtubule organization

Cells were plated on fibronectin-coated coverslips for 8 h before fixation and stained with tubulin antibody. The images were acquired by using a wide-field microscope. For the blind phenotypic analysis, the microtubule network was categorized into three groups based on their organization at the cell periphery: 1. ‘aligned’, 2. ‘intermediate’, 3. ‘disorganized’. The ‘intermediate’ organization was defined as where microtubule showed somewhat tangled or bended organization that was in between normal ‘aligned’ and abnormal ‘disorganized’ organization.

### Quantification of EB1 foci resident time on adhesions

Cells transfected with EGFP-EB1 and miRFP670-Paxillin were imaged at 1 s per frame for the duration of 1 min at TIRF plane using of Deltavision OMX SR (Cytiva) with 60×/1.49NA Apo N oil objective (Olympus). The channels for the obtained time-lapse series were aligned with SoftWoRx 7.0. The obtained time-lapse series were processed using FIJI/ImageJ. Different areas at the cell periphery were selected by using the ROI tool. The obtained time-lapse series of EB1 and Paxillin were processed by using the ‘manual tracking plugin’ in ImageJ. The resident time was calculated by tracking the number of frames between the first and last frame where an individual EB1 foci localized with a paxillin positive adhesion. The first frame is defined by the event where an individual EB1 foci comes in contact with an adhesion. Thereafter, all the consecutive frames were tracked where the particular EB1 foci stays stably associated with the adhesion until the last frame before it no longer localizes with the same adhesion. The individual EB1 foci were assigned to a specific track number from the start to end of the tracking. We specifically analyzed the events where EB1 foci co-localize with paxillin, whereas the events where EB1 foci seemed to slide off on the paxillin site or were present in the close proximity of paxillin site were not considered for the resident time analysis.

### Statistics

The statistical analysis and graph construction for intensity line profile (Fig. 1b, f; Supplementary Fig. 1a, b, c), for bar graph generation (Figs. 4c–e; 5b, e), co-sedimentation assay analysis (Fig. 7c) and all the FRAP data were performed with Excel (Microsoft). All the remaining graphs were constructed with OriginPro 2022b (OriginLab Corp.). Statistical tests were performed using two-tailed Student’s *t*-tests (Figs. 5f; 6b; Fig. 7b; Supplementary Fig. 6b, c, e; Fig. 4b, g; Supplementary Fig. 5b; Figs. 3e; 4g), Unpaired t test with Welch’s correction (Supplementary Fig. 6g). One-Way ANOVA followed by Tukey’s multiple comparison’s test (Fig. 3c, g, h; Supplementary 5f) and with two-tailed Mann–Whitney rank-sum tests (Supplementary Fig. 4g, h; Fig. 4h; Supplementary Fig. 6i) with OriginPro 2022b (OriginLab Corp.).

### Reporting summary

Further information on research design is available in the Nature Portfolio Reporting Summary linked to this article.

### Supplementary information

Supplementary Information

Peer Review File

Description of Additional Supplementary Files

Supplementary Movie 1

Supplementary Movie 2

Supplementary Movie 3

Supplementary Movie 4

Supplementary Movie 5

Supplementary Movie 6

Supplementary Movie 7

Supplementary Movie 8

Reporting Summary

### Source data

Source Data

## Data Availability

The data supporting the findings of the study are available in the manuscript and Supplementary Information file. Other raw data generated in the study are available from the corresponding author upon request. Source data are provided with this paper.
