# Supplementary material for: Focal adhesions contain three specialized actin nanoscale layers
Source: Nat Commun. 2024 Mar 21;15:2547. doi: 10.1038/s41467-024-46868-7 (PMC10957975; doi:10.1038/s41467-024-46868-7)
Supplement: Supplementary file 1 — Supplementary Information [file 41467_2024_46868_MOESM1_ESM.pdf]

SUPPLEMENTARY INFORMATION

Supplementary figures

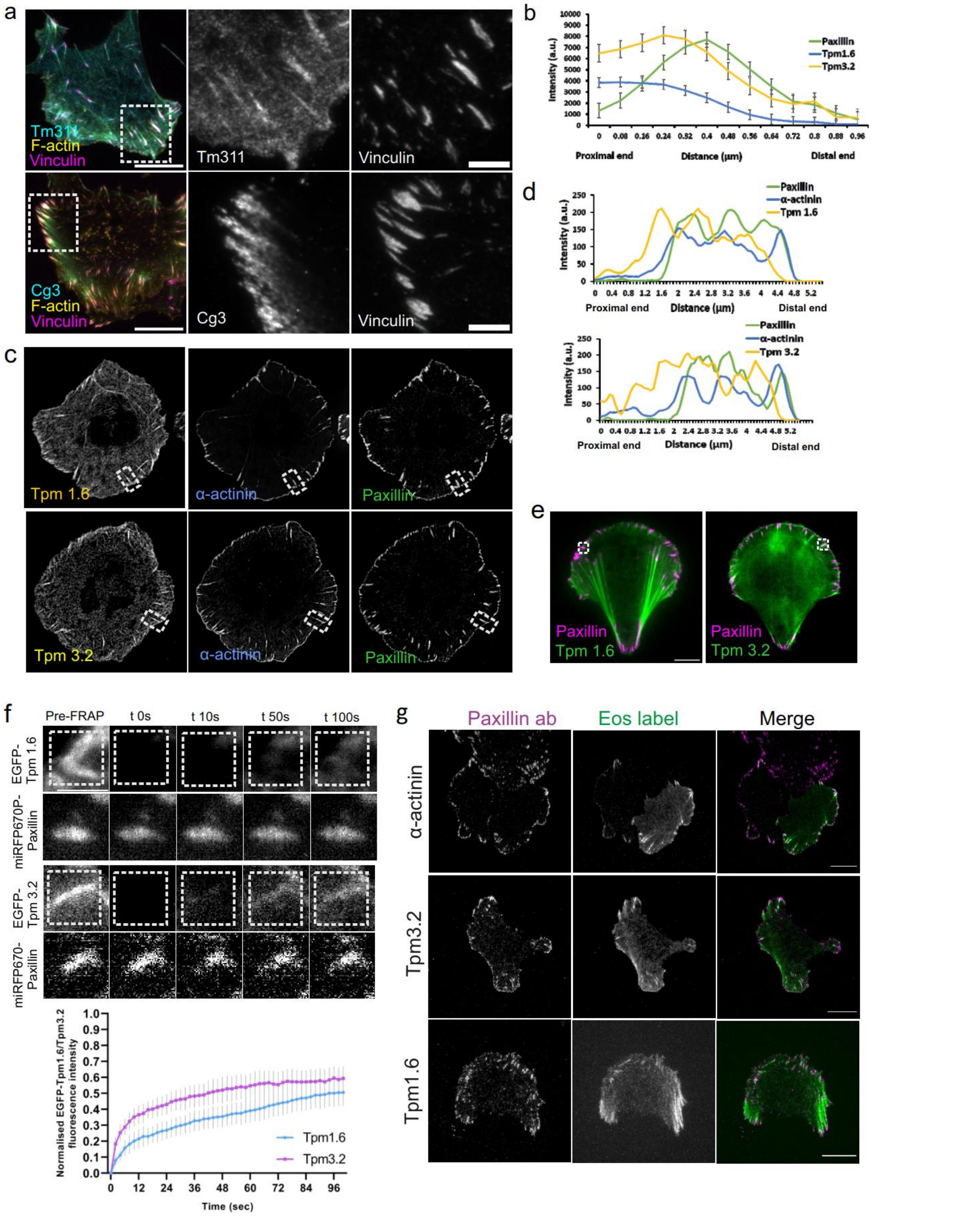

Supplementary Fig. 1. Localizations of Tpm1.6, Tpm3.2, α-actinin and paxillin in focal adhesions. (a) Representative TIRF images of wild-type U2OS cells stained for endogenous TM311 antibody (recognizing Tpm1 and Tpm2 splice variants), CG3 (recognizing Tpm3.1 and Tpm3.2), and with

vinculin-specific antibody. The panels on the right are enlarged views of the regions indicated by white boxes in the whole cell images on the left panels. Scale bars, 20 µm and 5 µm, respectively. (b) Intensity profile analysis, showing the lateral localizations of mRuby2C1-Tpm1.6 and pEGFPC1-Tpm3.2 from the proximal to distal end of focal adhesions of a wild-type U2OS cell (n=11 focal adhesions with the length of less than 1 µm from 5 cells). The quantification is related to the data presented in main figure 1a and b. The graph represents mean ± SE. (c) Representative TIRF images of wild-type U2OS cells expressing mRuby2C1-Tpm1.6 and EGFP-α-actinin, and stained for endogenous paxillin. Scale bars, 10 µm**.** (d) Examples of line-scan intensity profiles of the selected focal adhesions (indicated with white boxes in panel 'c'). (e) Representative time-lapse images from FRAP analyses on the dynamics of EGFP-Tpm1.6 and EGFP-Tpm3.2 in wild-type U2OS cells plated on crossbow micropatterns, where focal adhesions were also marked by co-expression of fluorescence fusion of paxillin. White boxes indicate photobleached regions. (f) Fluorescence recovery of EGFP-Tpm1.6 (cyan) and EGFP-Tpm3.2 (pink) in paxillin-positive adhesions (form panel 'e'). The graph below shows mean recovery curves of Tpm1.6 and Tpm3.2 ± S.D. over time. The measurements are from (n=19 adhesions from 6 movies) for Tpm1.6 and (n=20 adhesions from 5 movies) for Tpm3.2. (g) Validation of the photoconvertible constructs for iPALM imaging. Representative TIRF images of fixed U2OS cells expressing endogenous paxillin in combination with mEos3.2-α-actinin, mEos3.2-Tpm3.2, and mEos3.2-Tpm1.6. Scale bar 10 µm.

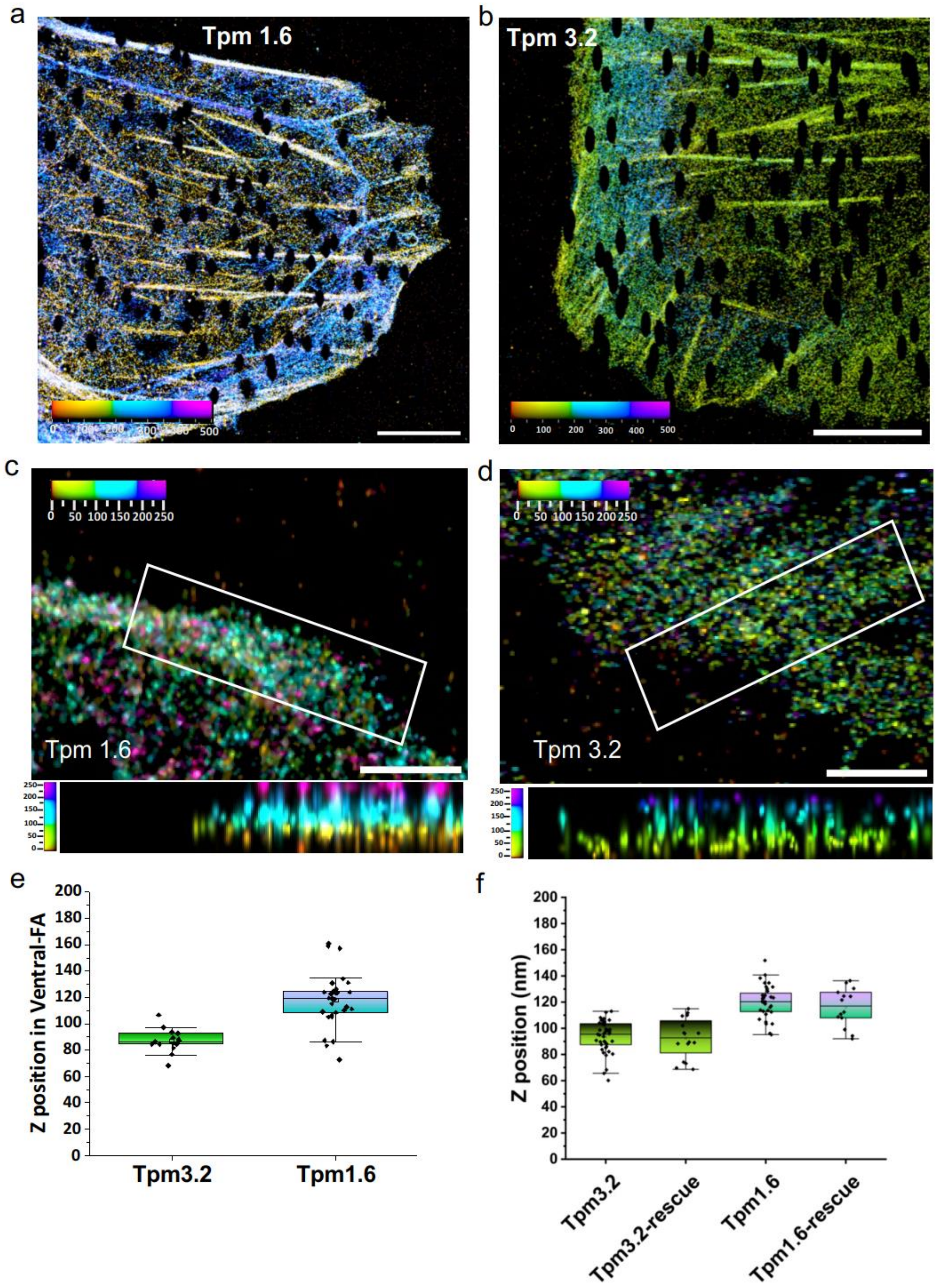

Supplementary Fig. 2. iPALM analysis of Tpm1.6 and Tpm3.2 localization in focal adhesions at the ends of ventral stress fibers. (a-b). Top views of rendered iPALM images with colours indicating the z coordinates from 0 to 500 nm, displaying the overall distribution of Tpm1.6 and Tpm3.2 in U2OS cells. Scale bars, 5 µm. Please note that the empty cell regions in the images represent the positions of fiducial beads, which were subtracted from the rendered images using a MATLAB code (for details, see 'Methods'). (c-d) Top views and side views (from regions indicated by white boxes in

top-view panels) from iPALM images of focal adhesions associated with ventral stress fibers. Scale bars, 2 µm. (e) Vertical Stratification of focal adhesions associated with ventral stress fibers showing the Z-positions (Z_{center}) of Tpm3.2 and Tpm1.6. Each point in the graph corresponds to an individual focal adhesion measurement. Boxes display the mean, median, Whiskers, IQR: 25th- 75th percentiles, Whiskers range within 1.5*IQR. (f) Vertical stratification of focal adhesions showing the Z-positions (Z_{center}) of Tpm3.2 and Tpm1.6 in the wild-type, as well as in the Tpm3 knockout (Tpm3.2-rescue), and Tpm1 knockout (Tpm1.6-rescue) cells. Each point in the graph corresponds to an individual focal adhesion measurement. Boxes display the mean, median, Whiskers, IQR: 25th- 75th percentiles, Whiskers range within 1.5*IQR.

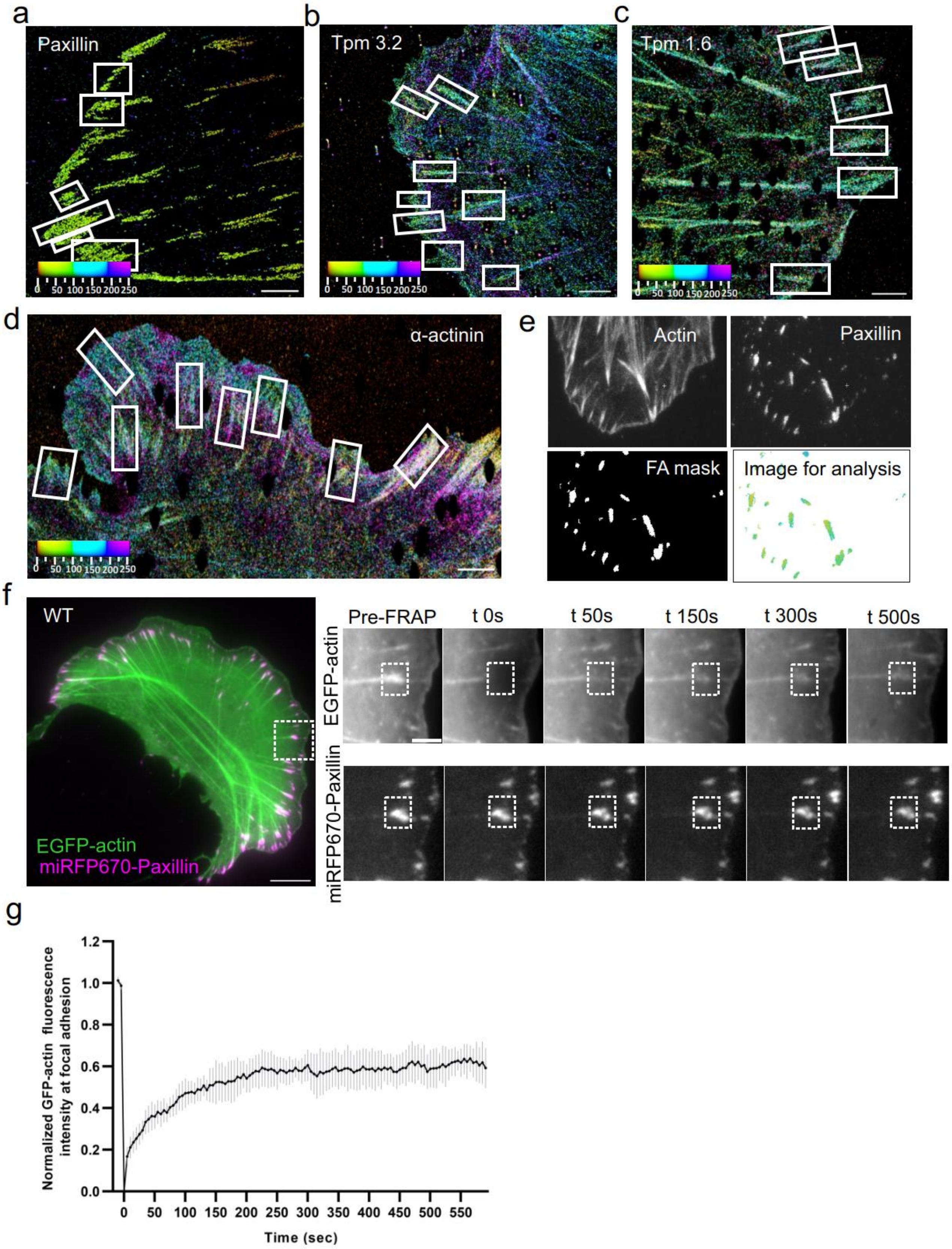

Supplementary Fig. 3. Analysis of focal adhesions located at the ends of dorsal stress fibers. Representative examples of top views of rendered iPALM images with colours indicating the Z-coordinates from 0 to 250 nm. The white boxes indicate the adhesions, which were used for quantifying the vertical stratification of individual proteins. (a) Endogenous paxillin. (b) mEos3.2-

Tpm3.2. (c) mEos3.2-Tpm1.6. (d) mEos3.2-α-actinin. Scale bars, 5 μm. (e) Example of focal adhesion mask generation using MATLAB and iPALM plotter (see Methods) that was applied for quantification of the Z-distribution of proteins in focal adhesions. (f) Representative examples of EGFP-actin and miRFP670-Paxillin time-lapse images (from a FRAP experiment) carried out on wild-type U2OS cells. The panels on the right are magnified images of the region indicated with a white box in the whole-cell image on the left and represent selected time-frames of the FRAP experiment. The time-point 'Pre' is the frame before bleaching and '0 s' is the first frame after bleaching. White boxes in the time-lapse images indicate the photo-bleached region. Scale bars, 10 μm and 3 μm in whole cell and time-lapse images, respectively. (d) Quantification of the fluorescence recovery of EGFP-actin at FAs of wild-type U2OS cells. Graph shows mean recovery curves ± S.D. over time. The measurements are from n=14 adhesions from 8 movies.

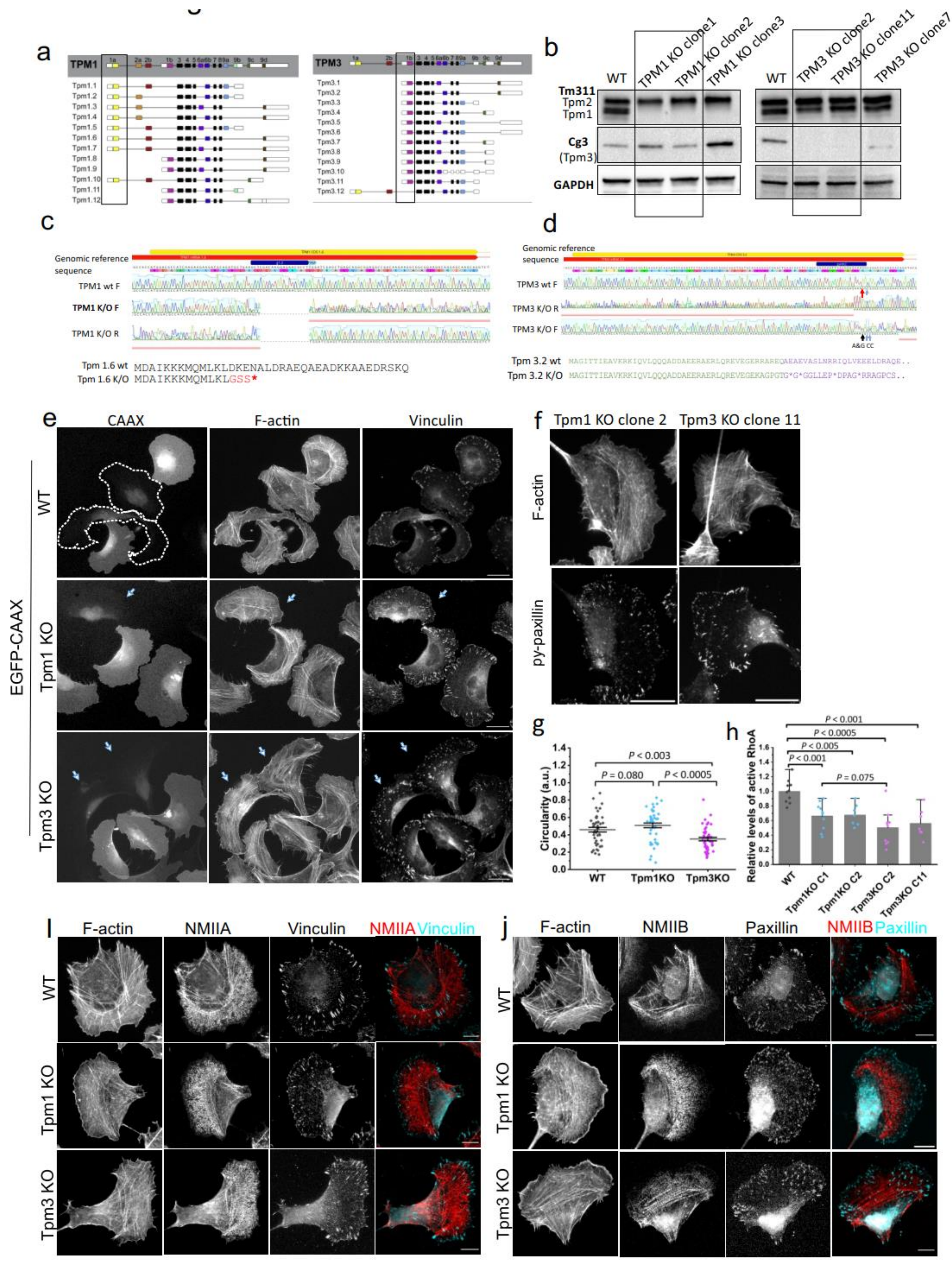

Supplementary Fig. 4. Generation and analysis of Tpm1 and Tpm3 knockout cells. (a). Schematic representation of the intron-exon organization of the mammalian *TPM1* and *TPM3* genes (adapted from Schevov et al., 2011 [ref 50]). The coloured boxes represent the protein coding exons. Black boxes indicate the specific exons targeted for generating CRISPR-Cas9 knockouts for Tpm1 and Tpm3

isoforms. (b) Western blot images of Tpm1 and Tpm3 protein levels in the lysates of wild-type, Tpm1 knockout and Tpm3 knockout cells, probed for antibodies against Tpm1 (TM311: recognizes products fromTpm1 and Tpm2) and Tpm3 (CG3: recognizes products from Tpm3 genes). GAPDH was probed for equal sample loading. (c-d) DNA sequences obtained from wild-type and two *TPM1* knockout (panel C) as well as wild-type and two *TPM3* (panel D) clones generated by CRISPRCas9 approach. Amino acid sequences below show how the mutations in the knockout clones lead to truncations of Tpm1.6 and Tpm3.2 proteins close to their N-termini. (e) Representative wide-field images of wild-type U2OS cells stably expressing EGFP-CAAX mixed with control wild-type U2OS cells, Tpm1 knockout and Tpm3 knockout cells, and stained for F-actin (phalloidin) and focal adhesions (vinculin antibody). The white dotted lines highlight the wild-type U2OS cells (not expressing EGFP-CAAX) in upper panel, and arrows highlight the Tpm1 and Tpm3 knockout cells in the lower panels. Scale bar, 20 µm. (f) Representative wide-field images of Tpm1 knockout clone 2, and Tpm3 knockout clone 11 plated on fibronectin-coated coverslips and stained for F-actin (phalloidin) and focal adhesion (phospho-paxillin antibody). Scale bar 20 µm. (g) Cell circularity analysis of wild-type (n=44), Tpm1 knockout clone 1 (n=46), and Tpm3 knockout clone 2 (n=46) cells after 8 hours of post-plating on fibronectin coated surface. The graph represents mean ± SE. (h) G-Lisa analysis of the levels of active RhoA in wild-type cells, Tpm1 knockout cells and Tpm3 knockout cells. Data are from three independent experiments and were normalized to the values of wild-type cells. The graph represents mean ± SE (i-j) Representative wide-field images of wild-type, Tpm1 knockout, and Tpm3 knockout U2OS cells stained for F-actin (phalloidin), focal adhesions (vinculin or paxillin antibodies), and with NMIIA and NMIIB antibodies. Scale bars, 10 µm.

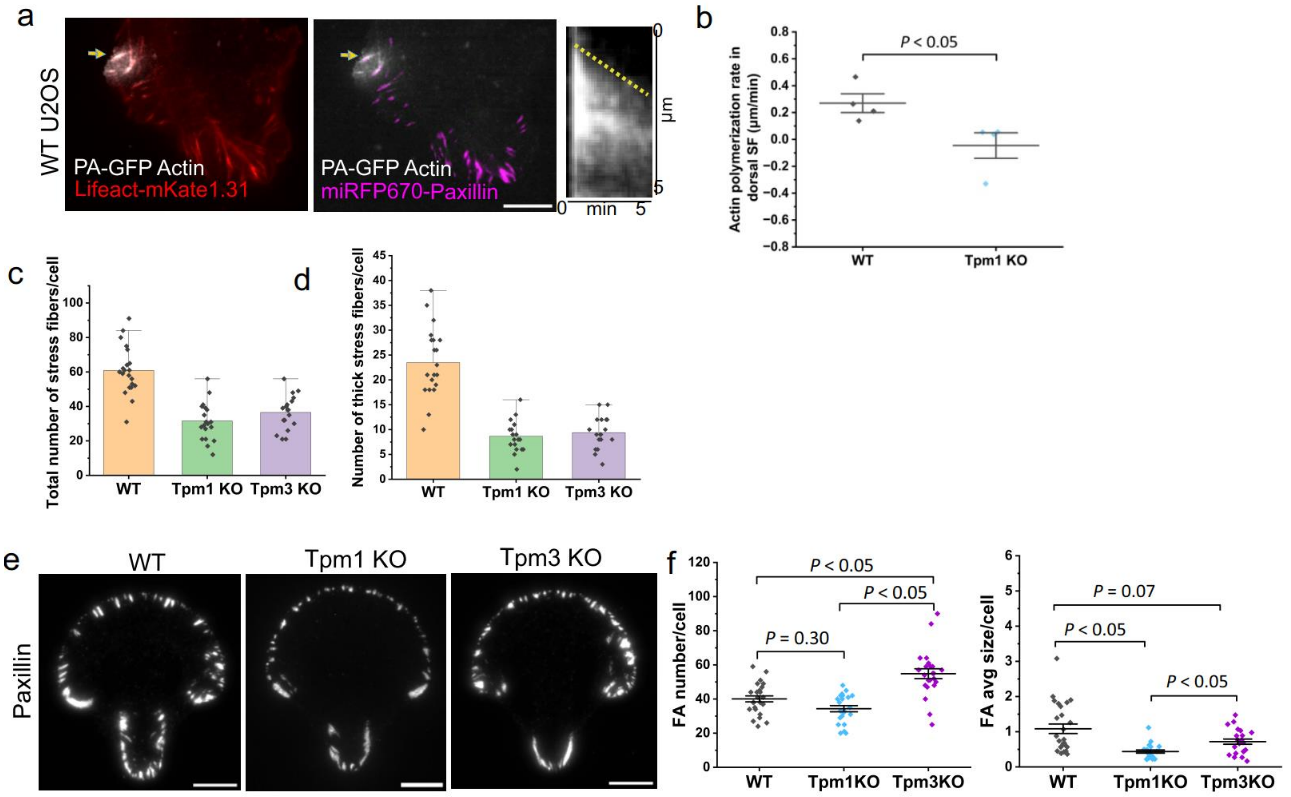

Supplementary Fig. 5. Analysis of actin assembly and stress fiber phenotypes in Tpm1 and Tpm3 knockout cells. (a) An example of photoactivation of PA-GFP-actin (gray) at the distal tip of a dorsal stress fiber in wild-type U2OS cell, which additionally expressed Lifeact-mKate1.31 (red) and

miRFP670-paxillin (magenta). PA-GFP-actin was activated adjacent to a paxillin-positive adhesion. Kymograph of PA-GFP-actin dynamics from the selected stress fiber indicated in the whole cell images is shown on right. Scale bar, 10 µm. (b) Quantification of the actin retrograde flow rates in dorsal stress fibers. Actin retrograde flow rates (µm/min) are shown as mean with ±S.E. Rates for individual data points were obtained from kymographs, subtracting the distance of the intensities at time points 10 s and 300 s after photoactivation. n = 4 foci (wild-type), and n=4 foci (Tpm1 knockout cells). (c-d) Analysis of the total numbers of stress fibers (panel c) and thick stress fiber bundles (panel d) in wild-type (n=24 cells), Tpm1 knockout (n=20 cells), and Tpm3 knockout cells (n=18 cells) by using ridge detection plugin in Fiji Image J software. The graph represents mean ± SE. (e) Examples of U2OS cells plated on crossbow micropatterns imaged with TIRFM. Focal adhesions were visualized by paxillin antibody. Scale bars, 10 µm. (f). Quantification of focal adhesion number and average size in cells under confinement (plated on crossbow micropatterns) demonstrating increased focal adhesion number in the Tpm3 knockout cells and decreased focal adhesion size in the Tpm1 knockout cells. These data suggest that the adhesion phenotypes in Tpm1 and Tpm3 knock-out cells are independent of cell migration. Data are from (n=26) wild-type, (n=22) Tpm1 knockout, and (n=23) Tpm3 knockout cells. The graph represents mean ± SE.

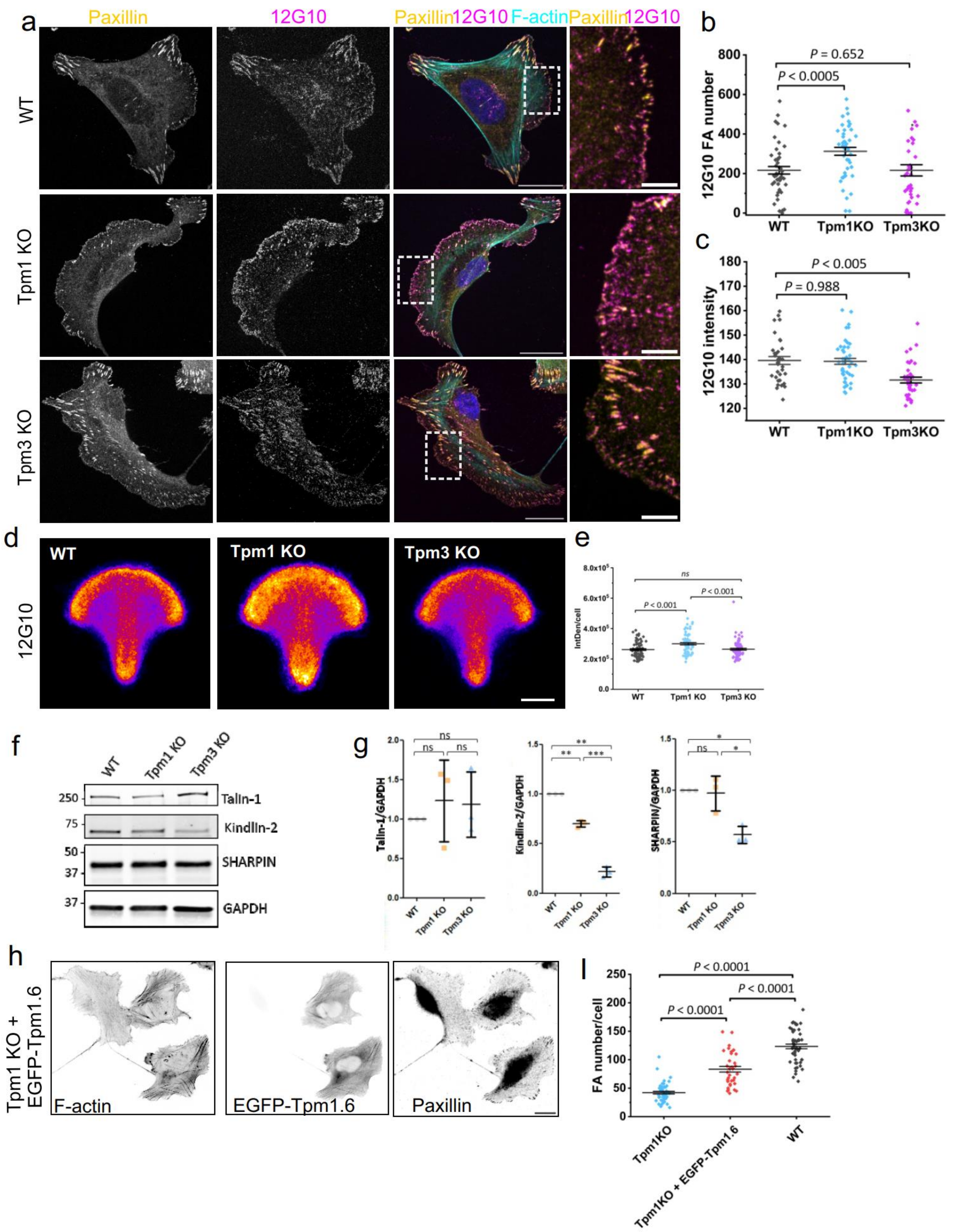

Supplementary Fig. 6. Effects of tropomyosin-1 and tropomyosin-3 depletions on integrin. (a) Representative confocal images of fixed wild-type, Tpm1 knockout, and Tpm3 knockout cells stained for focal adhesions (paxillin antibody), active ItgB1 (12G10), F-actin (phalloidin), and nucleus (DAPI). The panels on the right are enlarged views of the regions indicated by white boxes. Scale bar 20 µm and 5 µm, respectively. (b) Quantification of the number of active ItgB1 (12G10) positive adhesions

in wild-type (n=47), Tpm1 knockout (n=46), and Tpm3 knockout (n=37) cells. The graph represents mean ± SE. (c) Quantitative analysis of mean fluorescence intensity of active ItgB1 (12G10) in wild-type (n=33), Tpm1 knockout (n=46), and Tpm3 knockout (n=38) cells. The graph represents mean ± SE. (d) Mean intensity maps of active integrin β1 (12G10) at the ventral surface of wild-type, Tpm1 knockout and Tpm3 knockout cells plated onto FN-coated crossbow micropatterns. Blue, low intensity; yellow, high intensity. Scale bar, 10 µm. All cells are from one individual experiment. Wild-type, n = 24; Tpm1 KO, n = 23; Tpm3 KO, n = 20 cells. (e). Quantification of active integrin β1 (12G10 antibody) intensity (integrated density) in cells plated on FN-coated micropatterns. Data are from three individual experiments. Wild-type, n = 75; Tpm1 KO, n = 74; Tpm3 KO, n = 77 cells. Data represent mean +/- SD. (f) Representative Western blot showing the levels of talin-1, kindlin-2 and SHARPIN from whole cell lysates of wild-type, Tpm1 knockout and Tpm3 knockout U2OS cells. GAPDH used as loading control. (g) Quantification of talin-1, kindlin-2 and SHARPIN levels, as normalised to GAPDH. Data represent mean +/- SD. N = 3. *** = $p < 0.001$, ** = $p < 0.005$, * = $p < 0.05$, ns = non-significant. (h) Representative examples of Tpm1 knockout cells expressing EGFP-Tpm1.6. F-actin was visualized by phalloidin and focal adhesions by paxillin antibody. Scale bar, 20 µm. (i) Quantification of average focal adhesion numbers in Tpm1 knockout (n=50), Tpm1 knockout + EGFP-Tpm1.6 rescue (n=36), and wild-type (n= 52) cells. The graph represents mean ± SE.

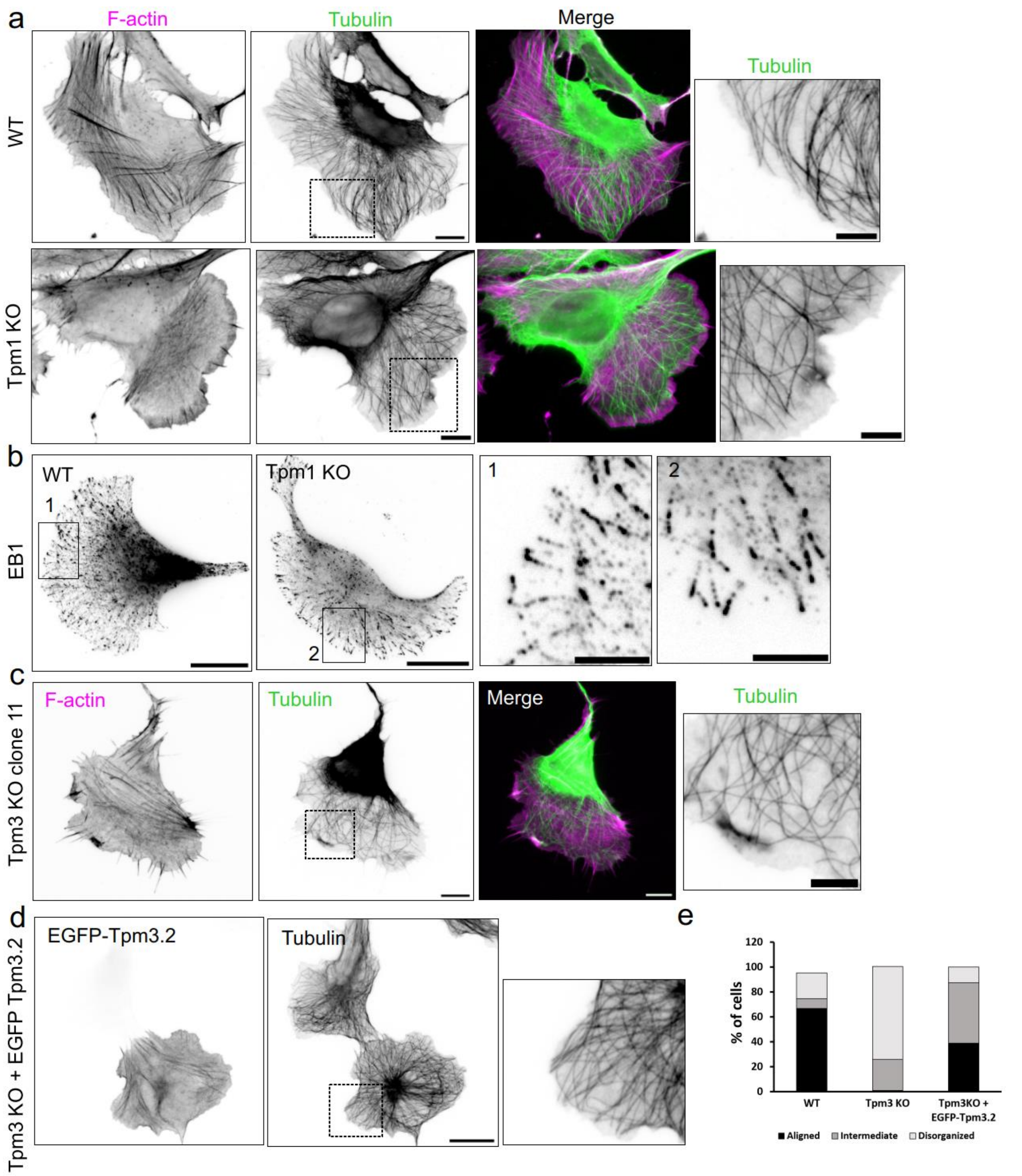

Supplementary Fig. 7. Effects of tropomyosin-1 depletion on microtubules. (a) Wide-field images of wild-type and Tpm1 knockout U2OS cells stained for F-actin (phalloidin) and microtubules (α-tubulin antibody). The panels on the right are magnified images of regions at the cell periphery (indicated with black boxes in the whole cell images). Scale bars, 10 µm and 5 µm, respectively. (b) Wide-field images of wild-type and Tpm1 knockout U2OS cells stained for EB1. The panels on the right (1-2) are magnified images of the regions indicated with black boxes in the whole cell images. Scale bars, 10 µm and 5 µm, respectively. (c) Representative example of Tpm3 knockout clone 11 cells stained for F-actin (phalloidin) and microtubules (α-tubulin antibody). The panels on the right are magnified images of regions at the cell periphery (indicated with black boxes in the whole cell

images). Scale bars, 10 µm and 5 µm, respectively. (d) Representative examples of Tpm3 knockout cells expressing EGFP-Tpm3.2. Microtubule networks were visualized with α-tubulin antibody. Scale bar, 20 µm. (e) Blind-analysis of percentage of cells displaying aligned, intermediate and tangled phenotypes of microtubule networks in wild-type (n= 63) and Tpm3 knockout (n=190), Tpm3 knockout + EGFP-Tpm3.2 rescue (n=72) cells.

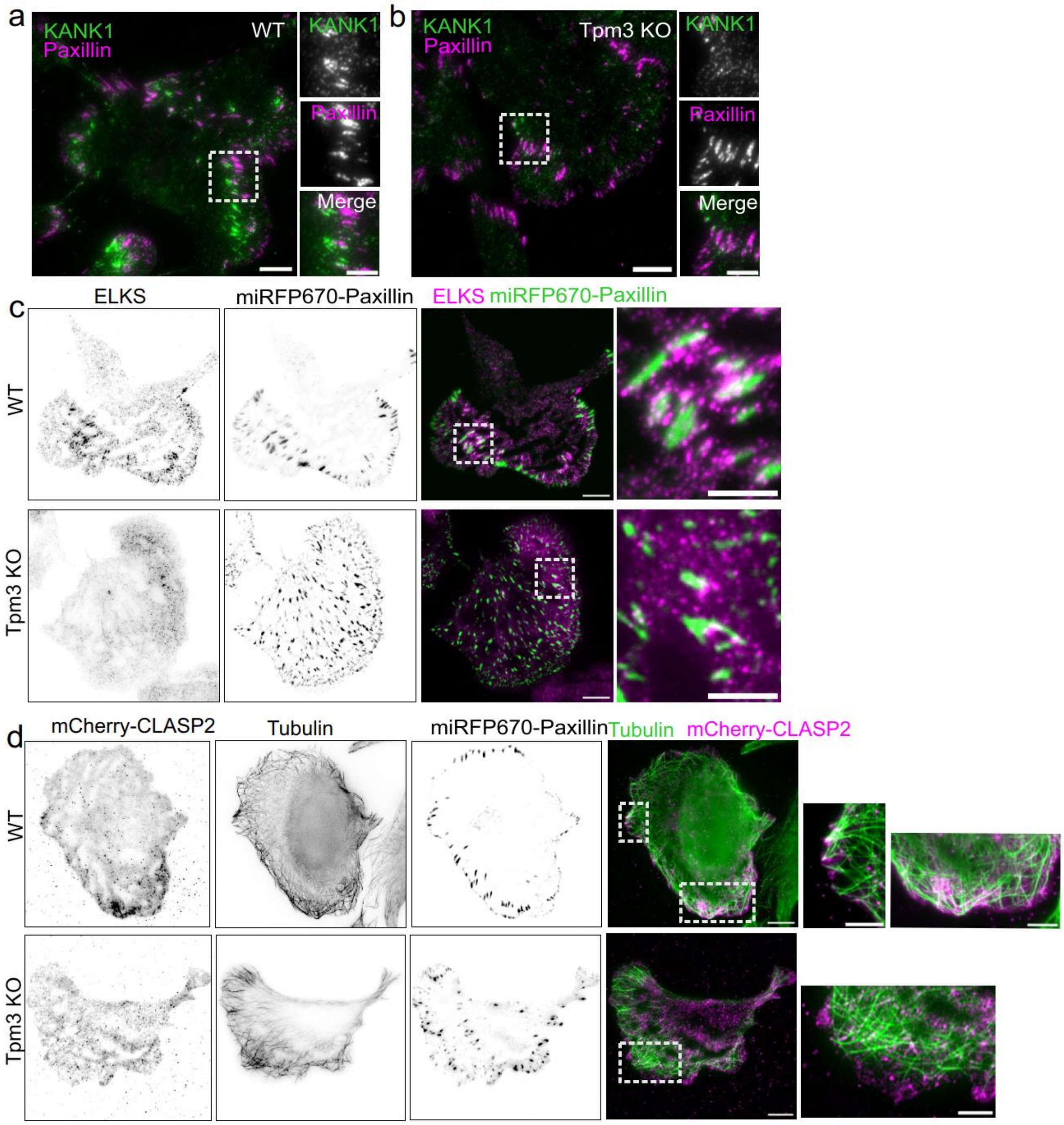

Supplementary Fig. 8. Effects of tropomyosin-3 depletion on the subcellular localizations of KANK-1, ELKS, and CLASP-2. (a-b). TIRF images of wild-type, Tpm3 knockout and Tpm1 knockout cells stained for endogenous KANK1 and paxillin. The panels on the right are magnified images of the regions indicated with white boxes in the whole cell images. Scale bars, 10 µm and 5 µm, respectively. (c) Representative semi-TIRF images of wild-type and Tpm3 knockout cells expressing miRFP670-Paxillin and where endogenous ELKS visualized by antibody staining. (d) Representative

semi-TIRF images of wild-type and Tpm3 knockout cells expressing mCherry-CLASP2, miRFP670-Paxillin, and stained with α-tubulin antibody. The panels at the right are magnified images of the regions indicated with white boxes in the whole cell images. Scale bars, 10 µm and 5 µm, respectively.

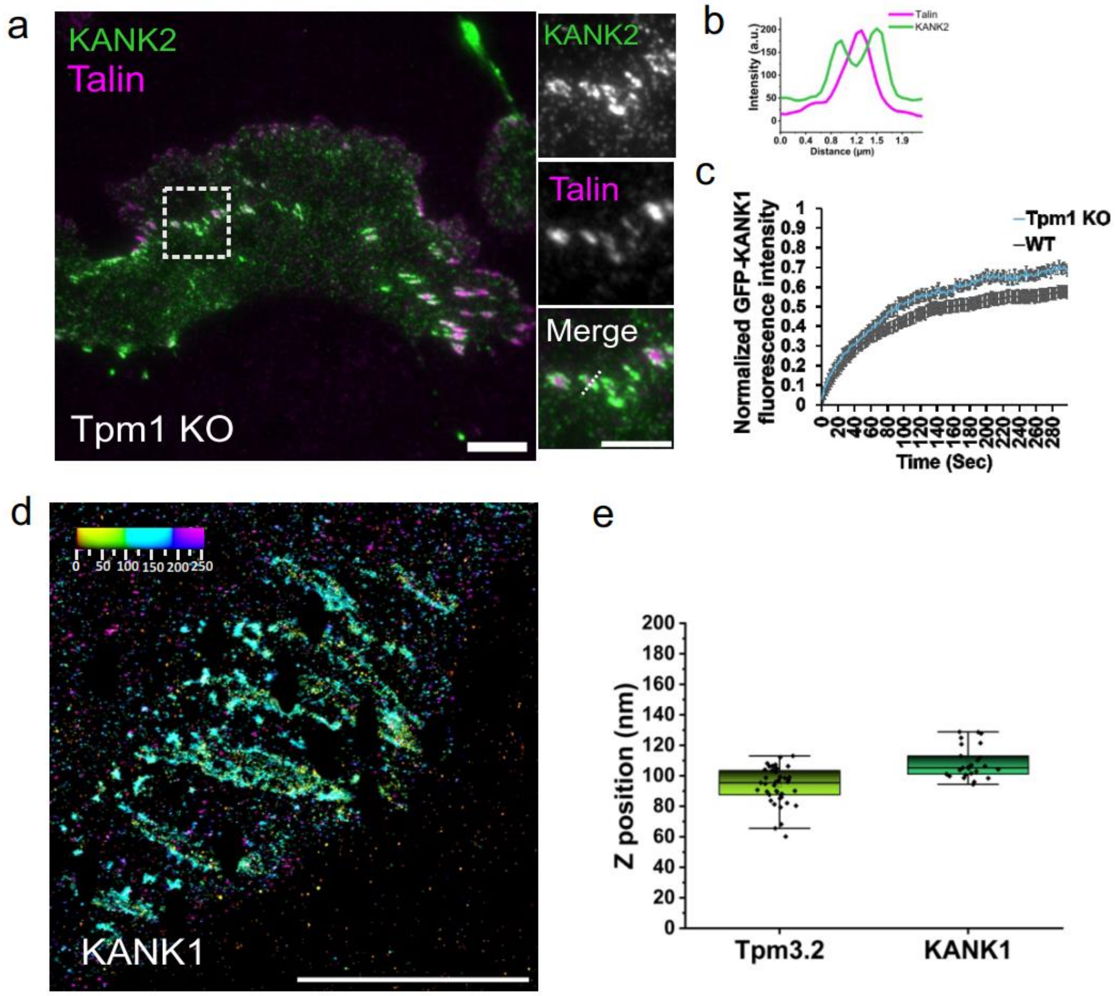

Supplementary Fig. 9. Effects of tropomyosin-1 depletion on the subcellular localizations of KANK2 and dynamics of KANK1. (a) TIRF images of Tpm1 knockout cells stained for endogenous KANK2 and talin. The panels on the right are magnified images of regions indicated with a white box in the whole cell image. (b) Intensity line scans from a selected region shown by the white dashed line in the magnified merge image. Scale bars, 10 µm and 5 µm, respectively. (c) Quantification of the fluorescence recovery of GFP-KANK1 in focal adhesions of wild-type and Tpm1 knockout cells. Graph shows mean curves ± S.D. over time. The measurements are from (n=29 KANK1 patches from 7 movies) wild-type and (n=18 KANK1 patches from 4 movies) Tpm1 knockout cells. (d) Top views of a rendered iPALM image with colours indicating the z coordinates from 0 to 250 nm, displaying the overall distribution of C-terminus of KANK1 in U2OS cells. Scale bars, 5 µm. (e) Vertical Stratification of focal adhesions showing the Z-positions (Z_{center}) of Tpm3.2 and C-terminus of KANK1 in wild-type cells. Each point in the graph corresponds to an individual focal adhesion measurement. Boxes display the mean, median, Whiskers, IQR: 25th- 75th percentiles, Whiskers range within 1.5*IQR.

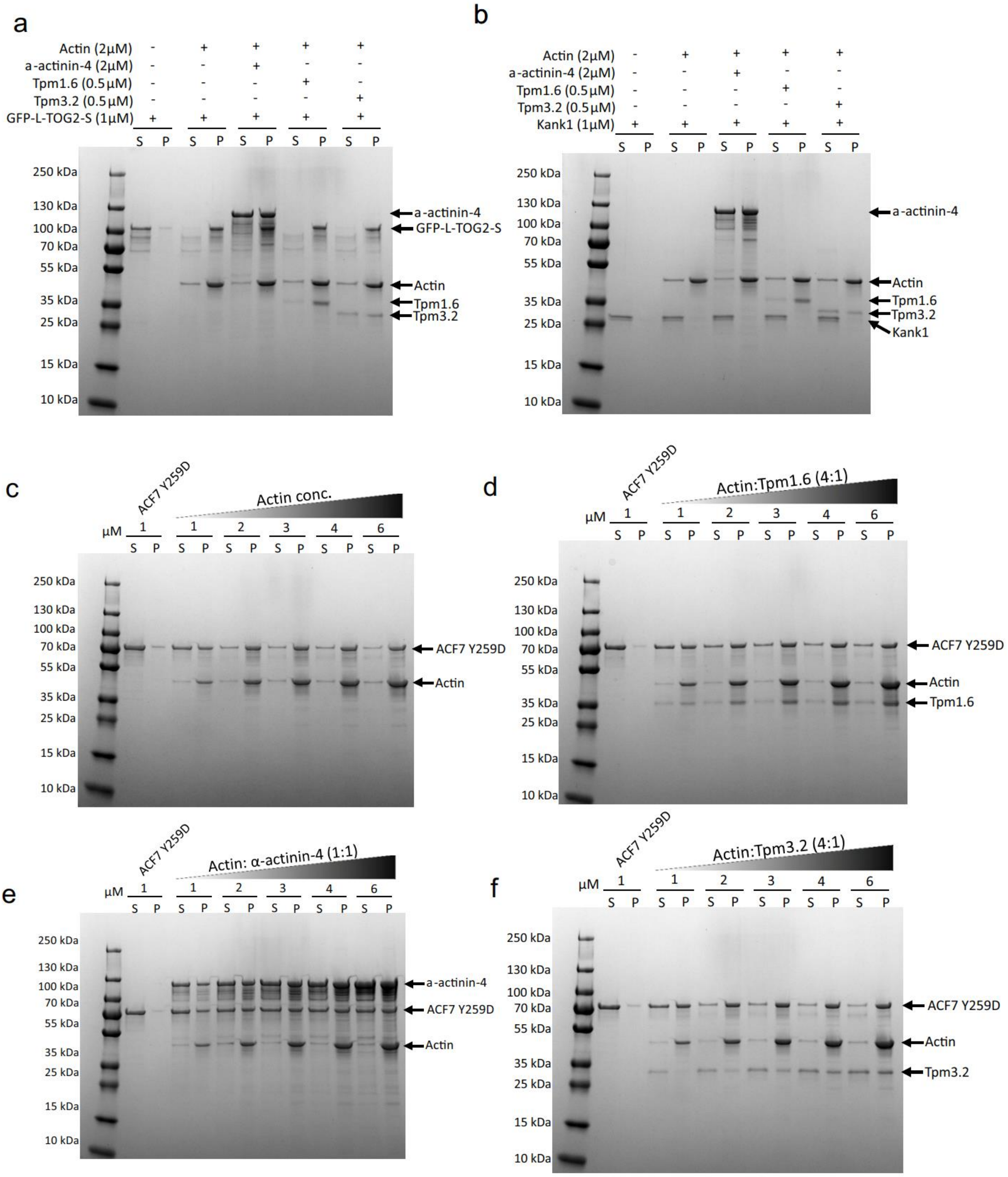

Supplementary Fig. 10. Interactions of KANK1, CLASP2, and ACF//MACF1 with actin filaments. (a-b) Representative gel images from co-sedimentation assays showing the binding of C-terminal ankyrin repeats of KANK1 and L-TOG2-S fragment of CLASP2 to bare actin filaments, and to actin filaments decorated with α-actinin-4, Tpm1.6 and Tpm3.2. Please note that CLASP2 L-TOG2-S fragment binds all four types of actin filaments with high affinity, whereas the KANK1 ankyrin repeat fragment does not display detectable binding to F-actin. (c-f) Representative gel images from actin filament co-sedimentation assays for ACF7 (residues 73-306, harboring an activating Y259D mutation) binding to bare actin filaments (panel c), and to actin filaments decorated with α-actinin-4 (panel d), Tpm1.6 (panel e) and Tpm3.2 (panel f).

Supplementary tables

Supplementary Table 1. iPALM statistics. Table containing the Z_{centre} values (averaged distance from the glass coverslip) of the proteins imaged with iPALM, number of molecule localizations, number of regions of interest, and number of cells used for analysis.

Protein	Average Z_{centre} + S.D. (nm)	# of localization	# of regions of interest	# of cells
Paxillin	51 ± 14.2	1.71E+05	28	3
Tpm3.2	93 ± 11.9	1.71E+05	41	6
Tpm1.6	120 ± 12.1	2.49E+05	36	4
α-actinin	139 ± 22.0	1.55E+05	55	6
actin	112 ± 21.9	2.52E+05	26	3
Kank1	108 ± 10.2	1.53E+05	25	3
Tpm3.2 in the background of Tpm3 knock-out cells	92 ± 14.14	0.32E+05	16	4
Tpm1.6 in the background of Tpm1 knock-out cells	116 ± 13.61	0.12E+05	14	3

Supplementary Table 2. Plasmids used in this study

Plasmids	Source	Additional information/comment
miRFP670-ggPaxillin	Lehtimaki et al., 2021	pPL1514
pEGFPC1-HsTpm1.6	Gateva et al., 2017	pPL1126
pEGFPC1-HsTpm3.2	Gateva et al., 2017	pPL1130
pmRuby2C1-HsTpm3.2	Gateva et al., 2017	pPL1136
GFP-alpha-actinin1	Kokate et al., 2022	pPL0285
mEos3.2-HsTpm1.6-FL	This study	pPL1239
mEos3.2-HsTpm3.2-FL	This study	pPL1241
mEos3.2-Hs-Alpha-Actinin1	Addgene # 57444	pPL1237
mEos3.2-KANK1	Stubb et al., 2019	
GFP-α-Tubulin	This study	pPL1279
pEGFP-EB1	Addgene # 17234	pPL1858
bioGFP-KANK1	A gift from Anna Akhmanova lab	pPL1866
GFP-KANK1-KN-L1	This study	pPL2043
mCherry-CLASP2	A gift from Anna Akhmanova lab	pPL1867
pRSETa-GFP-L-TOG2-S (261-793)	A gift from Ekaterina Grishchuk's Lab	pPL2014
pHis9-SUMO-HsKANK1(1073-1353)	This study	pPL2017
pHIS9-MBP-ACF7(73-306) Y259D	This study	pPL2015
pET-30a(+)-HsACTN4_FL	A gift from William Brieher (Cristian Suarez/ David Kovar)	pPL1993
LifeAct-mKate1.31	Addgene	Lehtimaki et al., 2021
PA-GFP-Actin	Addgene	Lehtimaki et al., 2021
LifeAct-TagGFP2	Addgene	Lehtimaki et al., 2021
EGFP-actin	Tojkander et al., 2015	pPL1389

Supplementary Table 3. Oligonucleotides used in this study

PCR product	Forward (5'-3')	Reverse (5'-3')
GFP vector from GFP-KANK1 (pPL1866)	TGAGTCGACGGTACCGCGGGC	AGAATTCGAAGCTTGAGCTCGAGATCT GAGTCCG
KN-L1-KANK1 domain insert from GFP-KANK1 (pPL1866)	TCTCGAGCTCAAGCTTCGAATTCTTACTT TGTGGAGACCCCCTATGGTTATCAAC	GCCCGCGGTACCGTCGACTCAGCTCACG TTGGTCACTGGGGTGG
KANK1-FL vector from GFP-KANK1 (pPL1866)	AGGCGGACCGGTCGCCACCATGGTGAG CAAGGGCGAGGAG	TTGAGCTCGAGATCTGAGTCCGGACTTG TACAGCTCGTCCATGCCGAG
paGFP insert from paGFP (pPL1831)	TCCGGACTCAGATCTCGAGCTCAAGCTT CG	GGTGGCGACCGGTCCGCCT
paGFP-KANK1 vector from GFP-KANK1 (pPL1866)	TGAGTCGACGGTACCGCGGGC	AGAATTCGAAGCTTGAGCTCGAGATCT GAGTCCG
pHis9-SUMO-HsKANK1(1073-1353)	TTCCAGCAGCAGACGGGAGGGAACCT GAGAAGGTGGAAATCAGAGAGAGG	TTTCGGGCTTTGTTAGCAGCCGGATCTC AATCAAATGAACCTCGGTGGGTGG
pHIS9-MBP-ACF7(73-306) Y259D	TTACGTTAGTAGTATCGACGATGCTTTC CCAAAGGTGCC	AAAGCATCGTCGATACTACTAACGTAAG TGATGACGCTCTTC
mEos3.2-HsTpm1.6-FL	EOS_F: TAACTGATCATAATCAGCCATACCACAT TTGTAG<br>Tpm1.6_F: AATGCCAGACGAGGATCCATGGCTTCTA TGGACGCC	EOS_R: ATGGATCCTCGTCTGGCATTGTCAGGCA ATC<br>Tpm1.6_R: GTGGTATGGCTGATTATGATCAGTTATC ACATGTTGTTTAACTCCAGTAAAGTCTG ATCC
mEos3.2-HsTpm3.2-FL	EOS_F: TAACTGATCATAATCAGCCATACCACAT TTGTAG<br>Tpm3.2_F: AATGCCAGACGAGGATCCATGGCTTCTA TGGCTGGG	EOS_R: ATGGATCCTCGTCTGGCATTGTCAGGCA ATC<br>Tpm3.2_R: GTGGTATGGCTGATTATGATCAGTTACT ACATCTCATTCAGGTCAAGCAGGGTC
