# Supplementary material for: Focal adhesions contain three specialized actin nanoscale layers
Source: Nat Commun. 2024 Mar 21;15:2547. doi: 10.1038/s41467-024-46868-7 (PMC10957975; doi:10.1038/s41467-024-46868-7)
Supplement: Supplementary file 3 — Description of Additional Supplementary Files [file 41467_2024_46868_MOESM3_ESM.pdf]

Description of Additional Supplementary Files

File Name: Supplementary Movie 1
Description: Tropomyosin recruitment to focal adhesions. Zoom in of a U2OS cell expressing miRFP670-Paxillin, EGFPTpm1.6 and mRuby2-Tpm3.2 migrating on a fibronectin (10 µg/ml)-coated high-precision imaging dish. Related to Figure 1. Time-lapse TIRFM video was recorded with ONI Nanoimager with 30 s imaging interval. Playback rate 10 frames/s. Scale bar 2 µm.

File Name: Supplementary Movie 2
Description: Nanoscale localizations of Tpm1.6 and Tpm3.2 in focal adhesions of U2OS cells. 3D reconstruction of iPALM data highlighting the slightly higher z distribution of mEos3.2-HsTpm1.6 compared to the z distribution of mEos3.2-HsTpm3.2. Related to Figure 1.

File Name: Supplementary Movie 3
Description: Random migration of wild-type, Tpm1 knockout and Tpm3 knockout U2OS cells on fibronectin. Time-lapse imaging example of wild-type, Tpm1 knockout, and Tpm3 knockout U2OS cells seeded on fibronectin coated (10 µg/ml)-12 well plate. Time-lapse images were captured by Cell-IQ (CM Technologies) with 8 min interval for 25 h. Please note the defects in tail retraction of Tpm3 knockout cells during migration. Related to Figure 3.

File Name: Supplementary Movie 4
Description: Focal adhesion dynamics in a wild-type cell. A wild-type U2OS cell expressing LifeAct-TagGFP2 (on the left) and miRFP670-Paxillin (on the right) migrating on a fibronectin (10 µg/ml)- coated high-precision imaging dish. Related to Figure 4. Time-lapse ring TIRFM video was recorded with Deltavision OMX SR with 30 s imaging interval. Playback rate 20 frames/s.

File Name: Supplementary Movie 5
Description: Focal adhesion dynamics in a Tpm1 knockout cell. A Tpm1 knockout cell expressing LifeAct-TagGFP2 (on the left) and miRFP670-Paxillin (on the right) migrating on a fibronectin (10 µg/ml)-coated high-precision imaging dish. Related to Figure 4. Time-lapse ring-TIRFM video was recorded with Deltavision OMX SR with 30 s imaging interval. Playback rate 20 frames/s.

File Name: Supplementary Movie 6
Description: Focal adhesion dynamics in a Tpm3 knockout cell. A Tpm3 knockout cell expressing LifeAct-TagGFP2 (on the left) and miRFP670-Paxillin (on the right) migrating on a fibronectin (10 µg/ml)-coated high-precision imaging dish. Related to Figure 4. Time-lapse ring-TIRFM video was recorded with Deltavision OMX SR with 30 s imaging interval. Playback rate 20 frames/s.

File Name: Supplementary Movie 7
Description: Dynamics of microtubules in wild-type and Tpm3 knockout cells. Zoom in of a wild type U2OS cell (on the left) and Tpm3 knockout cell (on the right) expressing miRFP670-Paxillin (magenta), GFP-α-tubulin (white) plated on a fibronectin (10 µg/ml)- coated high-precision imaging dish. Related to Figure 5. Time-lapse TIRFM video was recorded with Deltavision OMX SR with 3 s imaging interval. Playback rate 5 frames/s.

File Name: Supplementary Movie 8
Description: Live imaging of microtubule plus-end focal adhesion interplay in wildtype and Tpm3 knockout cells. Zoom in of a wild type U2OS cell (up) and Tpm3 knockout cell (bottom) expressing miRFP670-Paxillin, EGFPEB1 plated on a fibronectin (10 µg/ml)- coated high-precision imaging dish. Related to Figure 5. Time-lapse TIRFM video was recorded with Deltavision OMX SR with 3 s imaging interval. Playback rate 5 frames/s.
